# Automated synthesis and quality control of [^68^Ga]Ga-PentixaFor using the Gaia/Luna Elysia-Raytest module for CXCR4 PET imaging

**DOI:** 10.1186/s41181-023-00187-2

**Published:** 2023-02-07

**Authors:** Thomas Daniel, Clara Balouzet Ravinet, Jérôme Clerc, Rui Batista, Yvan Mouraeff

**Affiliations:** Cochin Hospital, Assistance Publique Hôpitaux de Paris, 123 Boulevard de Port Royal, 75014 Paris, France

**Keywords:** PET, PentixaFor, CXCR4, ^68^Ga, Automated synthesis, Radiopharmacy

## Abstract

**Background:**

[^68^Ga]Ga-PentixaFor is a promising radiotracer for positron emission tomography imaging of several human tumors overexpressing the chemokine receptor-4 (CXCR4). CXCR4 overexpression has been demonstrated in patients with hematologic malignancies, solid cancers, as well as cardiovascular pathologies of inflammatory origins. However, its radio synthesis is not yet fully developed in France, and existing methods do not use our type of synthesis module. Therefore, we aimed at developing a [^68^Ga]Ga-PentixaFor synthesis with Gaia/Luna Elysia-Raytest module to use it in clinical purpose.

**Results:**

12 syntheses were carried out by varying the temperature conditions and radiolabeling times, and led to choose specific labelling conditions with the Gaia/Luna Elysia-Raytest module: 97 °C, 4 min. The mean synthesis time of the 3 validation runs under good manufacturing practice (GMP) was 24 min 27 s (± 8 s), and the mean radiochemical yield was 87.0% [standard deviation (SD) 6.67%]. Different quality control parameters were also evaluated in accordance with European Pharmacopeia: radiochemical and radionuclidic purity, pH, sterility, stability and endotoxins levels. The average radiochemical purity was 99.1% (SD 0.25%) assessed by instant thin layer chromatography and 99.8% (SD 0.092%) assessed by high pressure liquid chromatography. average [^68^Ge] breakthrough was 1.48 × 10^–5^%, under the recommended level of 0.001%. We assessed the stability of the radiotracer up to 4 h at room temperature (no augmentation of the [^68^Ga] chloride in the final product, i.e. radiochemical purity (RCP) > 98.5%). The endotoxins levels were < 5 EU/mL, and the pH was 6.5 (same for the three syntheses).

**Conclusion:**

The [^68^Ga]Ga-PentixaFor synthesis process developed on the Gaia/Luna Elysia-Raytest module has fulfilled all acceptance criteria for injectable radiopharmaceutical products regarding the European Pharmacopeia. The radiochemical purity, stability, efficacy, as well as the microbiological quality of the three GMP batches were found to be good. The robustness of the synthesis process may be suitable for multi-dose application in clinical settings.

**Supplementary Information:**

The online version contains supplementary material available at 10.1186/s41181-023-00187-2.

## Background

The transmembrane chemokine receptor-4 (CXCR4) is involved in in embryogenesis, neoangiogenesis, hematopoiesis, and inflammation physiological process through the CXCR4/CXCL12 axis (Buck et al. 2022; Liu et al. 2021; Mousavi 2020). Nevertheless, its overexpression has been found to play a critical role in promoting tumor growth and progression, tumor invasiveness, and metastasis process (Balkwill 2004). Pathologies that can be found are mainly lymphoproliferative diseases such as B-cell lymphoma and lymphocytic leukemia, as well as cardiovascular pathologies of inflammatory origins (Kircher et al. 2018; Lapa et al. 2016; Mayerhoefer et al. 2018). In solid cancers the expression is more heterogeneous but is present in adrenal tumors, mammary cancer, prostate cancer or melanoma. In order to allow the evaluation and the follow-up of these pathologies, a radiotracer binding the CXCR4 receptor was developed for PET imaging (Demmer et al. 2011).

**Fig. 1:**
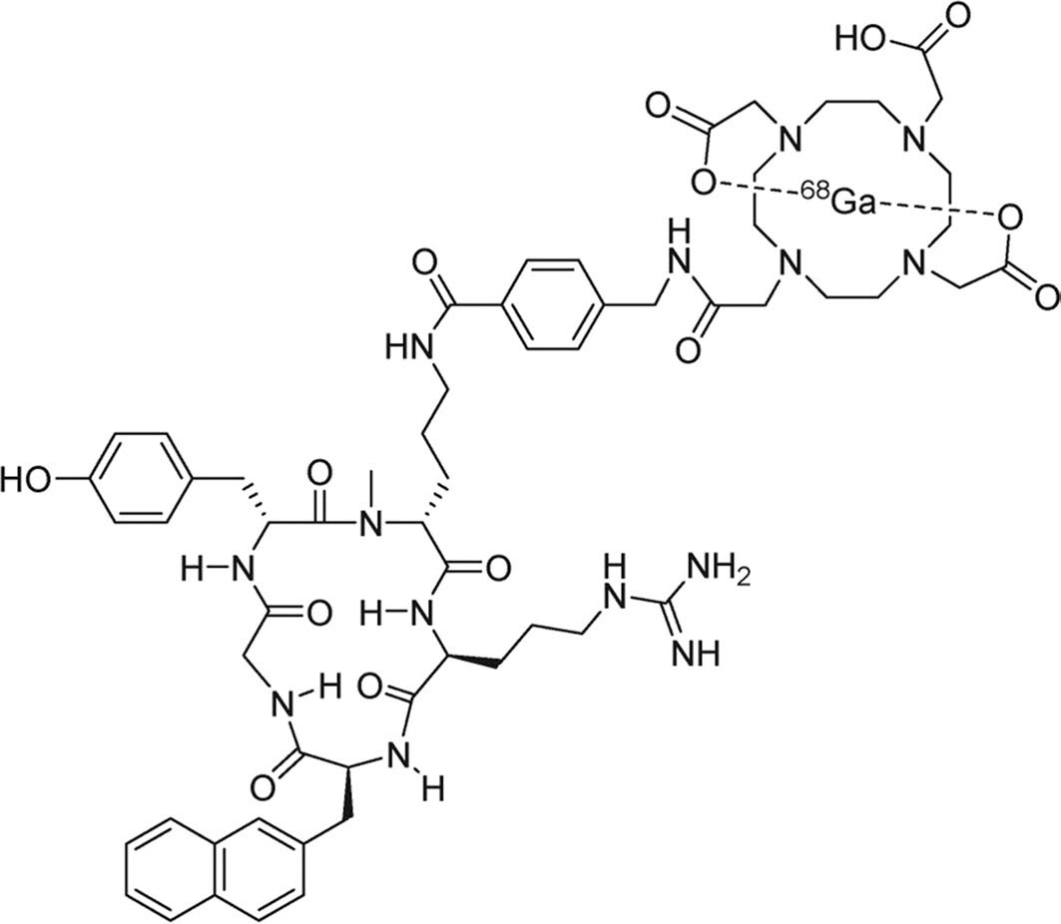
Chemical Structure depiction of [^68^Ga]Ga-PentixaFor (Schottelius et al. 2017)

[^68^Ga]Ga-PentixaFor (also known as C_60_H_77_GaN_14_O_14_ or ^68^Ga-CPCR4.2, Fig. 1 below) is thus a cyclic pentapeptide with high affinity to CXCR4, with suitable pharmacologic and pharmacodynamics characteristics, that make it an excellent PET imaging tracer (Demmer et al. 2011; Mayerhoefer et al. 2021; Schottelius et al. 2017). Its radiochemical synthesis has been developed and optimized to allow its use in clinical settings with different automated modules (Sammartano et al. 2020; Spreckelmeyer et al. 2020; Watts et al. 2021).

However, there is no description of a [^68^Ga]Ga-PentixaFor synthesis using the Gaia/Luna Elysia-Raytest module. Nevertheless, the type of module is important as it may affect the synthesis settings, such as the rise in temperature and the heating maintenance, or the synthesis duration. We thus decided to investigate and develop the radio synthesis of [^68^Ga]Ga-PentixaFor with the Gaia/Luna Elysia-Raytest module. First, we aimed at establishing the best conditions and synthesis sequences by testing a range of parameters and varying the commands of the module. Then, we aimed at carrying out GMP-grade syntheses, and performing pharmaceutical quality controls to validate the compatibility with the European Pharmacopeia. As there is no monography for [^68^Ga]Ga-PentixaFor in European pharmacopeia, we took as reference the monography of [^68^Ga]Ga-PSMA to validate our synthesis.

## Methods

### Reagents and devices

Good manufacturing process (GMP) grade PentixaFor and [^69^Ga]Ga-PentixaFor acetate were gracefully obtained from PentixaPharm GmbH (Würzburg, Germany).

The fluidic labelling kit for ^68^Ga labeling (sterile and single-use) and the reagents kit (including SCX and C18 cartridges, acetate buffer and HCl eluent solution, isotonic saline 0.9% solution, ethanol 60% and absolute ethanol solutions, water for injections, 0.22 µm filter and final vial—GMP grade) were purchased from ABX pharmaceuticals (*Advanced Biochemical Compounds*, Radeberg, Germany).

^68^Ga was obtained by elution of a commercial ^68^Ge/^68^Ga generator (GalliaPharm® 1850 MBq, Eckert & Ziegler radiopharma GmbH, Berlin, Germany) with a 0.1 M HCl solution (Eckert & Ziegler). The automated radio synthesis of [^68^Ga]Ga-PentixaFor was conducted on the commercial labeling synthesis module Gaia/Luna (Elysia-Raytest, GmbH, Straubenhardt, Germany). A computerized program was controlling the module, including the valves and syringes on the cassette, in order to produce the desired radiopharmaceutical.

### Description of the manufacturing process

Fifty micrograms of lyophilized precursor (PentixaFor) were dissolved with 2.2 mL of acetate buffer in a syringe, which was connected to the labeling cassette. The SCX and C18 cartridges were pre conditioned before elution with Water For injections (WFI), preceded for the C18 column by 5 mL of absolute ethanol, and dried. The peptide was then transferred into the reaction flask.

The elution of the generator was then carried out with 5 mL of 0.1 molar hydrochloric acid, and the ^68^Ga^3+^ was adsorbed on the cation exchange cartridge (SCX) in order to concentrate the radioactivity, evacuating the HCl 0.1 M in waste. The SCX column was then eluted with 0.5 ± 0.1 mL of eluent (WFI/NaCl 0.9%/HCl 32–35% mixture) directly in the reaction flask, with the peptide. This solution was heated at 97° Celsius during 4 min. The labeling was carried out at pH = 3.5.

**Fig. 2:**
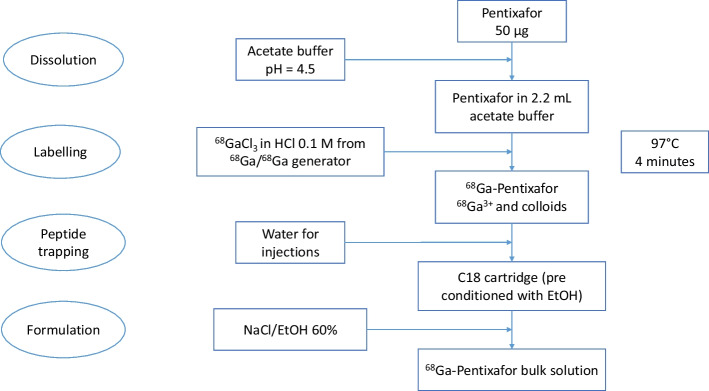
Flow-chart of the synthesis of the [^68^Ga]Ga-PentixaFor

At the end of the heating, the reaction mixture was transferred onto the C18 column, which retained the active substance ([^68^Ga]Ga-PentixaFor) as well as the colloidal forms (a radiochemical impurity that can occur during ^68^Ga labelling (Petrik et al. 2015; Velikyan 2015)), and evacuated the rest of the reaction mixture as waste (with the free gallium). The reaction flask was rinsed with WFI, and this solution was also transferred into the C18 column. The C18 column was then alternatively eluted with a mixture of 8.6 ± 0.3 mL of isotonic saline solution and 1.5 mL ± 0.1 mL of 60% ethanol, in order to elute the [^68^Ga]Ga-PentixaFor, while most of the colloidal forms remained on the column. The solution was finally transferred into the final vial through a 0.22 µm sterilizing filter (Millex-GV, 0.22 µm, hydrophilic, PVDF, 13 mm, sterilized with ethylene oxide) to obtain a volume of 10.1 mL ± 0.4 mL final solution (see Fig. 2).

### Adiochemical yield

The radiochemical yield was determined using the formula below (decay corrected):

$${\text{Radiochemical}}\;{\text{yield }} = \frac{{C18\;activity - C18\;Post\;Elution\;activity}}{{Initial\;activity\;measured\;in\;reactor}} \times 100$$

C18, C18 post elution and initial reactor activities were automatically measured and saved in the final software reports (available in Additional files 1, 2 and 3).

### Optimization of the synthesis settings

First tests were carried out to establish the command sequences of the synthesis module and find interesting ranges of temperatures and heating duration to investigate. Then, these different temperatures and heating times were tested in order to determine the optimal parameters for carrying out the radiolabeling, and to ensure the robustness of the process. A fixed quantity of precursor was used (50 μg). The ranges of settings tested were 95–98 °C and 4–6 min heating time, in agreement with the elements supplied by the PentixaPharm laboratory, the literature (Mueller et al. 2012; Spreckelmeyer et al. 2020; Watts et al. 2021) and our pre-tests, leading to the evaluation of 12 synthesis conditions.

For each setting, at least one synthesis was conducted and both radiochemical yield and radiochemical purity were determined. If several syntheses were carried out, we calculated the averages radiochemical yields and radiochemical purities.

### Validation and process controls of GMP-quality batches

We carried out several quality controls in order to check the accordance of the final product with the requirements for quality controls of radiopharmaceuticals (European Pharmacopeia).

#### Appearance and pH

We conducted a visual examination of each final product to verify the clearness and color of the solution: it had to be clear and colorless to comply the test. The pH-value of the solution was determined using pH strips. The expected pH was 5.0–8.0.

#### Chemical identity

We developed and validated a High-Pressure Liquid Chromatography (HPLC, Shimadzu, Kyoto, Japan) method in accordance with ICH Q2 standards. Briefly, we filled out a vial with a sample of the final product. An injection of 20 µL was done in a mobile phase in gradient mode (see Table 1 below), with a fixed flow rate of 1.0 mL/min during 15 min in a Phenomenex Gemini 3 m peptide NX-C188 110A LC 150 × 4.6 column (Torrance, USA). UV detection was performed at 220 nm with Diode Array Detector, and gamma detection was carried out with GABI Nova® radio flow monitor (NAI-Photomultiplier tube, Elysia-Raytest GmbH).

**Table 1:** Flow gradient used to perform HPLC analysis

HPLC analysis (flow = 1 mL/min)		
Time (min)	Mobile phase A (%) Water/TFA 1000/1 v/v	Mobile phase B (%) Acetonitrile/TFA 1000/1 v/v
0	80	20
9	50	50
10	50	50
15	80	20

Retention time of the final solution had to comply with reference material, constituted by the non-radioactive precursor ([^69^Ga]Ga-PentixaFor acetate). The retention time of [^68^Ga]Ga-PentixaFor solution had to be between 90 and 110% of that of [^69^Ga]Ga-PentixaFor acetate, taking into account that the UV and the gamma detectors were connected in series, so there was a fixed differential time of 15 s between the two analysis devices.

#### Radionuclidic identity

A gamma-spectrometry analysis was carried out on each batch with multi-channel-analyzer for gamma-spectroscopy MUCHA® (Elysia-Raytest GmbH) to measure the emitted energies. As ^68^Ga was the radionuclide of interest, we searched for the 511 keV (± 10%) main peak. Half-life was determined by automatized measuring of the final solution each second during 20 min. Result should be between 62 and 74 min (expected value of 67.71 min).

#### Radiochemical purity

Each batch of in-house prepared [^68^Ga]Ga-PentixaFor was tested to determine the RCP of the product, and part of impurities (free ^68^Ga and colloidal forms) due to the synthesis process.

We used an instant thin layer chromatography (ITLC) analysis to determine the part of free ^68^Ga and colloidal forms in the sample. Each component was characterized by its retention factor (R_f_). Rf is defined as the ratio of the distance traveled to the distance traveled by the solvent (solvent front). We used a sample of 10 µL taken directly from the final product to perform the ITLC analysis, with two methods:

- A first migration with a mobile phase constituted with 1 mol/L ammonium acetate/methanol (1:1) and as solid phase an ITLC-SG strip (Varian iTLC-SG plates): ^68^Ga colloidal forms and free ^68^Ga (i.e. ^68^Ga^3+^) were found with R_f_ = 0.0–0.2 (origin), and [^68^Ga]Ga-PentixaFor at R_f_ = 0.8–1
- A second migration with a mobile phase constituted with sodium citrate buffer (pH = 5.0) and as solid phase an ITLC-SG strip: [^68^Ga]Ga-PentixaFor and ^68^Ga colloidal forms were found with R_f_ = 0.0–0.2 (origin), and free ^68^Ga (^68^Ga.^3+^) at R_f_ = 0.8–1

A miniGITA® TLC scanning device (Elysia-Raytest GmbH) was used to achieve the ITLC analysis.

According to the European Pharmacopoeia, it is necessary to assess the radiochemical purity using HPLC. The method described in the chemical identity paragraph (Table 1) therefore allowed us to calculate the RCP by separating the [^68^Ga]Ga-PentixaFor from the free gallium (^68^Ga^3+^).

#### Radionuclidic purity

The ^68^Ge breakthrough was determined for each batch using multi-channel-analyzer for gamma-spectroscopy MUCHA® (Elysia-Raytest GmbH). 48 h after the synthesis (when all the presence of ^68^Ga is only due to the initial presence of ^68^Ge, as the half-life of ^68^Ga is 67.71 min), we measured the 511 keV peak emission due to the nuclear transformation of the ^68^Ge into ^68^Ga in a sample. Initial percentage of activity due to ^68^Ge was calculated as below:

$${\text{Germanium-68}}\;{\text{breakthrough}} = \frac{{Activity\;48\;h\;after\;synthesis\;\left( {decay\;corrected} \right)}}{{Activity\;measured\;in\;final\;vial\;just\;after\;synthesis}} \times 100$$

#### Bacterial endotoxins

A LAL-test using Endosafe® (Charles River, Wilmington, USA) device was conducted on each production, diluted to 1/100 with WFI endotoxin-free. This dilution level was chosen after determination of the maximal dilution, in order to avoid product interference with the test. The test was considered valid if the samples contained less than 15 EU/mL, in accordance with European Pharmacopeia requirements.

#### Radiochemical stability

In order to assess the stability of the preparations, we carried out a measurement of the RCP each hour during four hours after the syntheses, which may allow us to prepare the syringe for patients in clinical use regarding the half-life of ^68^Ga. The measurement was conducted using both iTLC and HPLC methods for RCP determination as previously specified.

## Results

### Final composition

At the end of the synthesis, the final vial contained an average of 614 MBq (SD 41.2 MBq) of [^68^Ga]Ga-PentixaFor, and so a specific activity of 12.3 MBq/µg (SD 0.82). The final volume of the preparation was 10.1 mL ± 0.4 mL. It contained 0.9 mL ± 0.06 mL of ethanol, 0.6 mL ± 0.04 mL of WFI and 8.6 mL ± 0.3 mL of sodium chloride, in accordance with the European Pharmacopeia requirements.

### Optimization of synthesis settings

The results of the syntheses conducted with different couple of settings (temperature/heating time) are presented below in Table 2.

**Table 2:** Radiochemical purity and radiochemical yield obtained with different synthesis settings

Temperature	Heating time	RCP	Radiochemical yield
95 °C	4	94.0%	85.9%
	5	94.7%	87.7%
	6	97.1%	76.2%
96 °C	4	93.8%	76.6%
	5	97.3%	88.2%
	6	95.8%	85.6%
**97 °C**	**4**	**98.4%**	**90.1%**
	5	97.1%	83.2%
	6	96.0%	77.4%
98 °C	4	97.6%	96.3%
	5	94.1%	79.3%
	6	94.6%	90.5%

Bold corresponds to the conditions finally chosen to conduct the radiosynthesis (GMP-quality batches)

Following these tests, we decided to carry out the GMP-grade syntheses at 97 °C for 4 min.

### Validation and quality control of GMP-quality batches

Three batches of [^68^Ga]Ga-PentixaFor (GMP grade) were produced, using the pre-determined parameters of synthesis (97 °C, 4 min). Complete synthesis reports can be found in 1, 2 and 3. Quality controls specifications and results are presented in Table 3:

**Table 3:** Results of GMP syntheses with regard to the specifications of the European Pharmacopeia

Tests	Methods	Specifications	Batch 1	Batch 2	Batch 3
Appearance	Visual	Clear, colorless solution	Complies	Complies	Complies
Identification: Photons γ emission peak	γ-Spectrometry (Ph. Eur. 2.2.66)	Principal peak at 0.511 MeV and 1.077 MeV, possible sum peak at 1.022 MeV	506 kEv	518 kEv	492 kEv
Period	Ionization chamber (Ph. Eur. 2.2.66)	62–74 min	66.3 min	69.3 min	64.4 min
pH value	pH strips (Ph. Eur. 4.1.1 1178900 and 2.2.4)	5.0 – 8.0	6.5	6.5	6.5
Sterility	Direct inoculation (Ph. Eur. 2.6.1 + 0125)	Sterile	Complies	Complies	Complies
Bacterial endotoxins	LAL-test (Ph. Eur. 2.6.14)	< 15 EU/mL	< 5 EU/mL	< 5 EU/mL	< 5 EU/mL
Radionuclidic purity ^68^Ga ^68^Ge and γ-emitted impurities	γ-Spectrometry (Ph. Eur. 2.2.66 + 0125)	≥ 99.9% ≤ 0.001%	Complies	Complies	Complies
Radiochemical purity [^68^Ga]Ga-PentixaFor	HPLC (Ph. Eur. 2.2.29)	≥ 95%	99.8%	99.8%	99.9%
^68^Ga^3+^chloride		≤ 2%	0.22%	0.24%	0.07%
[^68^Ga]Ga-PentixaFor	ITLC (Ph. Eur. 2.2.27)	≥ 95%	98.8%	99.2%	99.3%
^68^Ga^3+^chloride and ^68^Ga in colloidal forms		≤ 2%	0.42%	0.85%	0.20%
		≤ 3%	0.77%	0.56%	0.49%
Filter integrity test	Non-destructive test (Ph. Eur. 5.1.1)	Bubble point measurement > 3450 mbar)	Complies	Complies	Complies
Radioactivity	Ionization chamber (Ph. Eur. 2.2.66)	200–1400 MBq	586 MBq	661 MBq	594 MBq
Specific Activity	–	–	11.7 MBq/µg	13.2 MBq/µg	11.9 MBq/µg

All three batches fulfilled the requirements for injectable radiopharmaceutical products regarding the European Pharmacopeia.

### Stability assessment

**Fig. 3:**
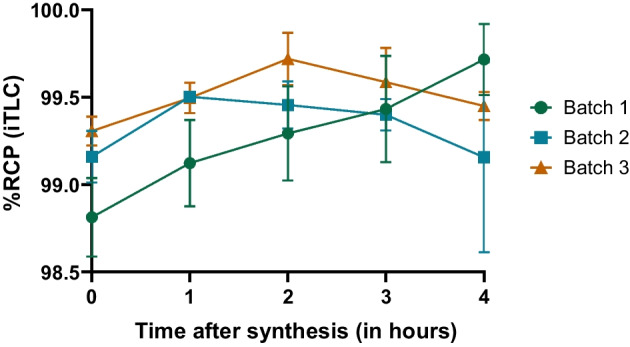
Time course of RCP of [^68^Ga]Ga-Pentixafor during stability test assessed by iTLC

**Fig. 4:**
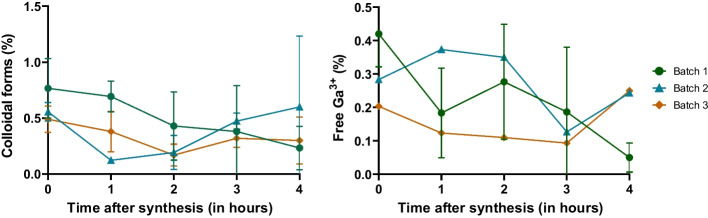
Time course of impurities (colloidal forms and free Ga^3+^ percentages) during stability test, assessed by iTLC

**Fig. 5:**
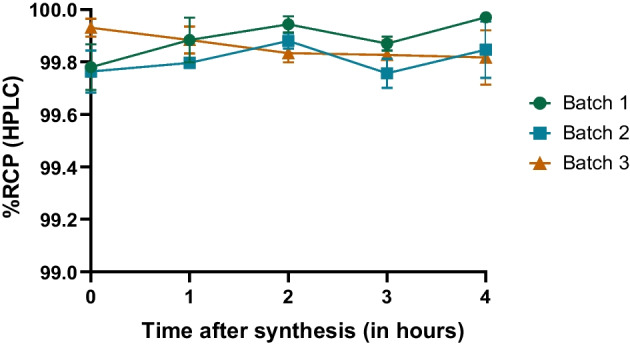
Time course of RCP of [^68^Ga]Ga-Pentixafor during stability test, assessed by HPLC

We performed both iTLC and HPLC methods in order to to determine the stability of the final product. Free Ga^3+^ and, if applicable, colloidal forms measurements were carried out every hour during 4 h in triplicate tests, as shown in Tables 4 and 5, and Figs. 3, 4 and 5 below.

**Table 4:** Radiochemical purity assessed using iTLC method during 4 h

RCP using iTLC method				
		Batch 1	Batch 2	Batch 3
Final vial radioactivity		586 MBq	661 MBq	594 MBq
T = 0 h	RCP	99.00%	99.30%	99.40%
		98.80%	99.10%	99.20%
		98.60%	99.10%	99.30%
	Colloidal forms	0.57%	0.53%	0.39%
		0.66%	0.65%	0.46%
		1.07%	0.49%	0.62%
	Free Ga^3+^	0.39%	0.14%	0.21%
		0.53%	0.26%	0.30%
		0.34%	0.45%	0.10%
T = 1 h	RCP	99.40%	99.50%	99.60%
		99.10%	99.50%	99.40%
		98.90%	99.50%	99.50%
	Colloidal forms	0.57%	0.12%	0.23%
		0.67%	0.13%	0.58%
		0.84%	0.12%	0.33%
	Free Ga^3+^	0.03%	0.37%	0.20%
		0.28%	0.36%	0.02%
		0.24%	0.39%	0.15%
T = 2 h	RCP	99.60%	99.60%	99.70%
		99.00%	99.30%	99.60%
		99.30%	99.50%	99.90%
	Colloidal forms	0.08%	0.06%	0.20%
		0.57%	0.36%	0.25%
		0.64%	0.16%	0.06%
	Free Ga^3+^	0.35%	0.35%	0.14%
		0.40%	0.32%	0.14%
		0.08%	0.38%	0.05%
T = 3 h	RCP	99.10%	99.40%	99.70%
		99.70%	99.50%	99.40%
		99.50%	99.30%	99.70%
	Colloidal forms	0.84%	0.41%	0.29%
		0.25%	0.46%	0.41%
		0.05%	0.55%	0.26%
	Free Ga^3+^	0.07%	0.19%	0.02%
		0.08%	0.05%	0.23%
		0.41%	0.14%	0.03%
T = 4 h	RCP	100%	98.50%	99.50%
		99.70%	99.40%	99.40%
		99.60%	99.50%	99.50%
	Colloidal forms	0.02%	1.27%	0.21%
		0.28%	0.52%	0.54%
		0.40%	0.01%	0.15%
	Free Ga^3+^	0.03%	0.20%	0.26%
		0.10%	0.04%	0.09%
		0.02%	0.49%	0.40%

**Table 5:** Radiochemical purity assessed using HPLC method during 4 h

RCP using HPLC method				
		Batch 1	Batch 2	Batch 3
Final vial radioactivity		586 MBq	661 MBq	594 MBq
T = 0 h	RCP	99.70%	99.80%	100%
		99.80%	99.80%	100%
		99.80%	99.70%	99.90%
	Free Ga^3+^	0.32%	0.23%	0.05%
		0.18%	0.16%	0.05%
		0.16%	0.32%	0.11%
T = 1 h	RCP	99.90%	99.80%	99.90%
		99.80%	99.80%	99.90%
		100%	99.80%	99.80%
	Free Ga^3+^	0.15%	0.20%	0.06%
		0.18%	0.19%	0.13%
		0.03%	0.22%	0.16%
T = 2 h	RCP	99.90%	99.90%	99.80%
		100%	99.90%	99.80%
		100%	99.90%	99.90%
	Free Ga^3+^	0.09%	0.09%	0.17%
		0.05%	0.15%	0.20%
		0.03%	0.12%	0.13%
T = 3 h	RCP	99.90%	99.80%	99.80%
		99.90%	99.70%	99.90%
		99.90%	99.80%	99.80%
	Free Ga^3+^	0.10%	0.24%	0.17%
		0.15%	0.30%	0.12%
		0.14%	0.19%	0.23%
T = 4 h	RCP	100%	99.90%	99.90%
		100%	99.70%	99.90%
		100%	99.90%	99.70%
	Free Ga^3+^	0.02%	0.13%	0.10%
		0.05%	0.27%	0.15%
		0.02%	0.06%	0.30%

All three batches were considered stable as their RCP proved to be superior to 98.5% during 4 h after the syntheses.

## Discussion

There are already several radio syntheses of [^68^Ga]Ga-PentixaFor described in the literature, using the Scintomics GRP Automated Synthesis Module (Scintomics GmbH) (Sammartano et al. 2020; Watts et al. 2021), or the Modular Lab PharmTracer (Eckert & Ziegler) (Spreckelmeyer et al. 2020). However, there are none describing the synthesis using the Gaia/Luna Elysia-Raystest module to our knowledge. The advantage of using this module consists in particular in the concentration of the radioactivity in the SCX cartridge at the beginning of the synthesis, which allows to control the volume of the reaction mixture.

Moreover, the radiochemical yields reported in the literature were found to be around 70–80%, which is somewhat lower than what we obtained with our process (average radiochemical yield of 87.0%, n = 3). However, we calculated the yield from in-process measurements to take into account the radioactivity actually transferred to the reaction vial and the results should therefore be compared with caution. As it was not possible to measure the activity eluted from the generator before introducing it to the fluidic labelling kit, calculations could also have been made taking into account the radioactivity in the final product and the theoretical value of ^68^Ga eluted from the generator, but the latter may vary slightly due to the variation in performance of elution during each synthesis.

The average radiochemical purity was 99.1% (n = 3), which is comparable with the existing literature previously cited, but this needs to be confirmed by a larger number of batches. These syntheses are sufficient in regard with the European Pharmacopoeia to ensure a reliable production method, and could be increased in the future.

As it is, our synthesis method is suitable for clinical use, fulfilling all European Pharmacopeia requirements dealing with injectable radiopharmaceutical drugs. In the near future, the synthesis process may be used in a clinical human trial in our hospital, and later for diagnostic purposes. For example, [^68^Ga]Ga-PentixaFor could become an interesting radiotracer in the determination of the laterality of aldosterone secretion in patients with primary aldosteronism (Cui et al. 2019). In this indication, PET imaging could become an interesting non-invasive tool versus the current gold-standard protocol, which consists in an adrenal vein sampling performance (Baz et al. 2022). Moreover, in parallel with labeling with gallium 68, this vector can be labelled with lutetium 177, which allow the development of theragnostic with this molecule (Herrmann et al. 2016; Maurer et al. 2019).

## Conclusion

The [^68^Ga]-PentixaFor synthesis process that we had developed on the Gaia/Luna Elysia-Raytest module showed a high radiochemical purity (> 98%) with a good radiolabelling efficacy (> 85%). The radiotracer can be considered as chemically stable during 4 h after the synthesis, which is suitable for clinical use in routine. The microbiological quality is also good and allows injection in human being. The synthesis fulfilled all acceptance criteria for injectable radiopharmaceutical products regarding the European Pharmacopeia. After validation by the competent health authorities, the [^68^Ga]Ga-PentixaFor may be used in our hospital to assess the laterality of aldosterone secretion in patients with primary aldosteronism, or in hematopoietic malignancies.

## Supplementary Information

**Additional file 1.** GMP-conditions synthesis report N°1. This document shows all details dealing with technique parameters of the Gaia/Luna Elysia-Raytest module during the first GMP-condition synthesis.

**Additional file 2.** GMP-conditions synthesis report N°2. This document shows all details dealing with technique parameters of the Gaia/Luna Elysia-Raytest module during the second GMP-condition synthesis.

**Additional file 3.**. GMP-conditions synthesis report N°3. This document shows all details dealing with technique parameters of the Gaia/Luna Elysia-Raytest module during the third GMP-condition synthesis.

## Data Availability

The datasets generated or analyzed during the current study are included in this published article. Supplementary information is available from the corresponding author on reasonable request.

## References

[CR1] Balkwill F (2004). Cancer and the chemokine network. Nat Rev Cancer.

[CR2] Baz AHC, van de Wiel E, Groenewoud H (2022). CXCR4-directed [^68^Ga]Ga-PentixaFor PET/CT versus adrenal vein sampling performance: a study protocol for a randomised two-step controlled diagnoStic Trial Ultimately comparing hypertenSion outcome in primary aldosteronism (CASTUS). BMJ Open.

[CR3] Buck AK, Haug A, Dreher N (2022). Imaging of C-X-C motif chemokine receptor 4 expression in 690 patients with solid or hematologic neoplasms using ^68^Ga-pentixafor PET. J Nucl Med.

[CR4] Cui Y, Zhang Y, Ding J (2019). A rare aldosterone-producing adenoma detected by ^68^Ga-pentixafor PET-CT: a case report and literature review. Front Endocrinol.

[CR5] Demmer O, Gourni E, Schumacher U, Kessler H, Wester HJ (2011). PET imaging of CXCR4 receptors in cancer by a new optimized ligand. ChemMedChem.

[CR6] Herrmann K, Schottelius M, Lapa C (2016). First-in-human experience of CXCR4-directed endoradiotherapy with 177Lu- and 90Y-labeled pentixather in advanced-stage multiple myeloma with extensive intra- and extramedullary disease. J Nucl Med.

[CR7] Kircher M, Herhaus P, Schottelius M (2018). CXCR4-directed theranostics in oncology and inflammation. Ann Nucl Med.

[CR8] Lapa C, Lückerath K, Kleinlein I (2016). ^68^Ga-Pentixafor-PET/CT for Imaging of chemokine receptor 4 expression in glioblastoma. Theranostics.

[CR9] Liu P, Sun H, Zhou X (2021). CXCL12/CXCR4 axis as a key mediator in atrial fibrillation via bioinformatics analysis and functional identification. Cell Death Dis.

[CR10] Maurer S, Herhaus P, Lippenmeyer R (2019). Side effects of CXC-chemokine receptor 4–directed endoradiotherapy with pentixather before hematopoietic stem cell transplantation. J Nucl Med.

[CR11] Mayerhoefer ME, Jaeger U, Staber P (2018). [^68^Ga]Ga-pentixafor PET/MRI for CXCR4 imaging of chronic lymphocytic leukemia: preliminary results. Invest Radiol.

[CR12] Mayerhoefer ME, Raderer M, Lamm W (2021). CXCR4 PET imaging of mantle cell lymphoma using [^68^Ga]Pentixafor: comparison with [18F]FDG-PET. Theranostics.

[CR13] Mousavi A (2020). CXCL12/CXCR4 signal transduction in diseases and its molecular approaches in targeted-therapy. Immunol Lett.

[CR14] Mueller D, Klette I, Baum RP, Gottschaldt M, Schultz MK, Breeman WAP (2012). Simplified NaCl based ^68^Ga concentration and labeling procedure for rapid synthesis of ^68^Ga radiopharmaceuticals in high radiochemical purity. Bioconjug Chem.

[CR15] Petrik M, Vlckova A, Novy Z, Urbanek L, Haas H, Decristoforo C (2015). Selected ^68^Ga-siderophores versus ^68^Ga-colloid and ^68^Ga-citrate: biodistribution and small animal imaging in mice. Biomed Pap Med Fac Univ Palacky Olomouc Czechoslov.

[CR16] Sammartano A, Migliari S, Scarlattei M, Baldari G, Ruffini L (2020). Synthesis, validation and quality controls of [^68^Ga]-DOTA-Pentixafor for PET imaging of chemokine receptor CXCR4 expression. Acta Bio Medica Atenei Parm.

[CR17] Schottelius M, Osl T, Poschenrieder A (2017). [177Lu]pentixather: comprehensive preclinical characterization of a first CXCR4-directed endoradiotherapeutic agent. Theranostics.

[CR18] Spreckelmeyer S, Schulze O, Brenner W (2020). Fully-automated production of [^68^Ga]Ga-PentixaFor on the module modular lab-pharmtracer. EJNMMI Radiopharm Chem.

[CR19] Velikyan I (2015). ^68^Ga-Based radiopharmaceuticals: production and application relationship. Mol Basel Switz.

[CR20] Watts A, Chutani S, Arora D (2021). Automated radiosynthesis, quality control, and biodistribution of Ga-^68^ pentixafor: first Indian experience. Indian J Nucl Med.

