# Supplementary material for: Automated synthesis and quality control of [68Ga]Ga-PentixaFor using the Gaia/Luna Elysia-Raytest module for CXCR4 PET imaging
Source: EJNMMI Radiopharm Chem. 2023 Feb 7;8:4. doi: 10.1186/s41181-023-00187-2 (PMC9905377; doi:10.1186/s41181-023-00187-2)

Rapport de synthèse

No lot:	PTX GMP 1
Méthode:	Pentixafor Rev. 71 (01/09/2022 09:21:19)
No kit:	01A1-2SH1-USQQ-DKE5-426QRT-101 RT-101-220510002-1 1
No peptide:	9916.0000.050/005 PenFor-05-20110901.01 1
Logiciel :	Gaia Control Rev. 2.2
Système:	Gaia: raytest Gaia, Numéro de série 35701, Rev. 1.30<br>Luna: raytest Luna, Numéro de série 35704, Rev. 1.04
Opérateur:	mouraeff (mouraeff)
Code kit:	Utilisé en R&D ou pour évaluation. Non valide en utilisation GMP!
Temps départ:	02/09/2022 09:59:39
Temps fin:	02/09/2022 10:24:09
Statut:	Synthèse complète

Préparation POS

✓ Mettre des gants stériles
✓ Resserer tous les raccords du kit fluidique
✓ Préparer le cartouche C18 SEPACK avec 5 ml d'éthanol absolu dans le petit becher
✓ Fixer la rampe A et installer la cartouche SCX entre A2 et B1 terminal
✓ Fixer la rampe B et installer la cartouche C18 entre B5 terminal et C2
✓ Fixer la rampe C
✓ Mettre le flacon de réaction sur le kit dans le four du GAIA
✓ Brancher la tubulure VYGON en A5 et la connecter au filtre 0,22 µm spécifique
✓ Connecter un aiguille STERICAN au filtre et l'inserer dans le flacon final de réaction
✓ Inserer une prise d'air 0,22 µm dans le flacon final de réaction
✓ Déconnecter le filtre 1,2 µm de la prise d'air B braun et connecter le filtre en A4
✓ Connecter la tubulure en A1 à l'embout de pression
✓ Positionner l'aiguille sterican jaune sur la tubulure en A1 terminal
✓ Inserer cette aiguille dans le flacon de dechet et inserer un prise d'air
✓ Faire des aller/retour avec le piston de la seringue d'éluant
✓ Connecter la seringue d'éluant C1 et la positionner sur la potence à droite
✓ Connecter la tubulure en C4 au flacon d'eau ppi et le positionner sur le support à droite
✓ Insérer la tubulure en C5 terminal dans la pompe perilstatique et le détécteur
✓ Connecter la tubulure en C5 à la sortie du générateur
✓ Connecter le flacon de NaCl 0,9 % (bleu) en B4 bleu
✓ Connecter le flacon d' ethanol 60 % (or) en B5 or
✓ Prélever 2,2mL de tampon à l'aide d'une seringue de 5mL et d'une aiguille micropine
✓ Connecter une aiguille sterican à cette seringue et prelever la totalité du tampon
✓ Ajuster la seringue et la connecter en B3
✓ Ouvrir les robinets de la poche de HCl 0,1 M dans le sens de l'élution

Résultats

Etape 25:	SCX Elution: Preparation:	942 MBq
Etape 29:	Labeling / Measuring Reactor Activity:	964 MBq
Etape 39:	C18: Purge / Measuring Empty Reaktor:	30 MBq
Etape 40:	C18: Purge / Measuring C18:	682 MBq
Etape 49:	Formulation: Purge / Measuring C18 Post_Elution:	50 MBq
Etape 50:	Venting Preparation / Measuring SCX Post_Elution:	26 MBq
Etape 59:	Filter Integrity Test: Measuring Bubble Point Value:	3866 mbar

Rendement:	67%
Durée de la synthèse:	0:09:49 (589s)

Journal de la synthèse

02/09/2022 09:59:39	Synthèse démarrée
+00:15	Etape 1 terminée: C18 Purge
+01:27	Etape 2 terminée: Kit Integrity Test: Pressurizing

+01:32	Etape 3 terminée: Kit Integrity Test: Equilibration
+01:47	Etape 4 terminée: Kit Integrity Test: Measuring
+01:52	Etape 5 terminée: Kit Integrity Test: Venting
+02:03	Etape 6 terminée: NaCl Syringe Preparation
+02:18	Etape 7 terminée: Buffer Addition: Preparation
+02:20	Etape 8 terminée: Buffer Addition: Preparation Venting
+02:50	Etape 9 terminée: Buffer Addition
+02:53	Etape 10 terminée: Buffer Addition: Venting
+03:23	Etape 11 terminée: Cartridge Activation: SCX + C18
+03:33	Etape 12 terminée: Cartridge Activation: C18
+03:38	Etape 13 terminée: Cartridge Activation: Purge Preparation
+03:43	Etape 14 terminée: Cartridge Activation: Purge 1
+03:54	Etape 15 terminée: Cartridge Activation: Purge 2
+04:04	Etape 16 terminée: Cartridge Activation: Purge 3
+04:07	Etape 17 terminée: Cartridge Activation: Venting
+06:17	Etape 18 terminée: Generator Elution: Collect
+06:27	Etape 19 terminée: Generator Elution: Waste
+06:37	Etape 20 terminée: SCX Washing: Line
+06:47	Etape 21 terminée: SCX Washing
+06:52	Etape 22 terminée: SCX Washing: Line Purge Preparation
+07:08	Etape 23 terminée: SCX Washing: Line Purge
+07:18	Etape 24 terminée: SCX Washing: Cartridge Purge
+07:30	Etape 25 terminée: SCX Elution: Preparation
+08:30	Etape 26 terminée: SCX Elution 1
+09:30	Etape 27 terminée: SCX Elution 2
+09:45	Etape 28 terminée: SCX Elution: Purge
+09:50	Etape 29 terminée: Labeling / Measuring Reactor Activity
+11:50	Etape 30 terminée: Labeling Part 1
+12:00	Etape 31 terminée: Labeling / Lift Purge
+13:51	Etape 32 terminée: Labeling Part 2
+14:03	Etape 33 terminée: Delution
+14:13	Etape 34 terminée: C18 Peptide Trapping: Preparation
+15:28	Etape 35 terminée: C18 Peptide Trapping
+15:46	Etape 36 terminée: Washing Reaktor
+15:57	Etape 37 terminée: C18 Washing: Preparation
+16:57	Etape 38 terminée: C18 Washing
+17:02	Etape 39 terminée: C18: Purge / Measuring Empty Reaktor
+17:07	Etape 40 terminée: C18: Purge / Measuring C18
+17:17	Etape 41 terminée: C18: Elution: EtOH
+17:27	Etape 42 terminée: C18: Elution: Water
+17:37	Etape 43 terminée: C18: Elution: EtOH
+17:52	Etape 44 terminée: C18: Elution: Water
+18:12	Etape 45 terminée: C18: Elution: EtOH
+18:32	Etape 46 terminée: C18: Elution: Water
+18:52	Etape 47 terminée: C18: Elution: EtOH
+20:23	Etape 48 terminée: Formulation
+20:43	Etape 49 terminée: Formulation: Purge / Measuring C18 Post_Elution
+20:48	Etape 50 terminée: Venting Preparation / Measuring SCX Post_Elution
+21:51	Etape 51 terminée: Remove Product, Connect Waste
+22:21	Etape 52 terminée: Filter Integrity Test: Filter Purge
+22:26	Etape 53 terminée: Filter Integrity Test: Line Purge Preparation
+22:41	Etape 54 terminée: Filter Integrity Test: Line Purge 1
+22:56	Etape 55 terminée: Filter Integrity Test: Line Purge 2
+23:06	Etape 56 terminée: Filter Integrity Test: Line Purge 3
+23:13	Etape 57 terminée: Filter Integrity Test: Pressurizing
+23:22	Etape 58 terminée: Filter Integrity Test: Approaching Bubble Point Value
+24:25	Etape 59 terminée: Filter Integrity Test: Measuring Bubble Point Value
+24:30	Etape 60 terminée: Venting
+24:30	Synthèse complète: 02/09/2022 10:24:09

Gaia–Vitesse de la pompe

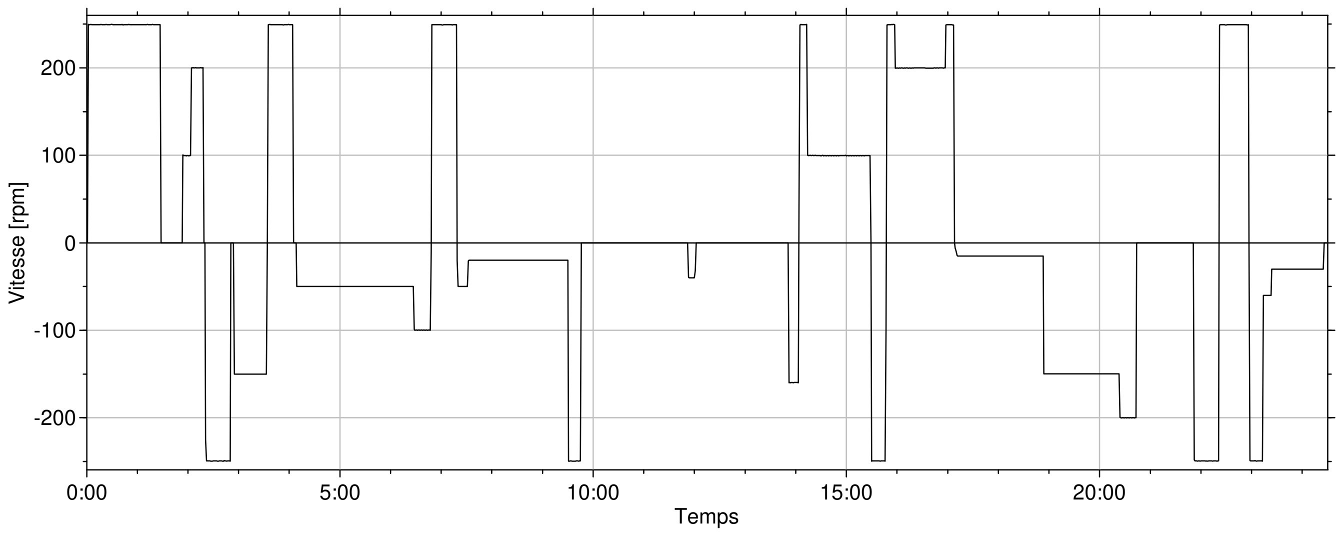

Gaia–Température du four

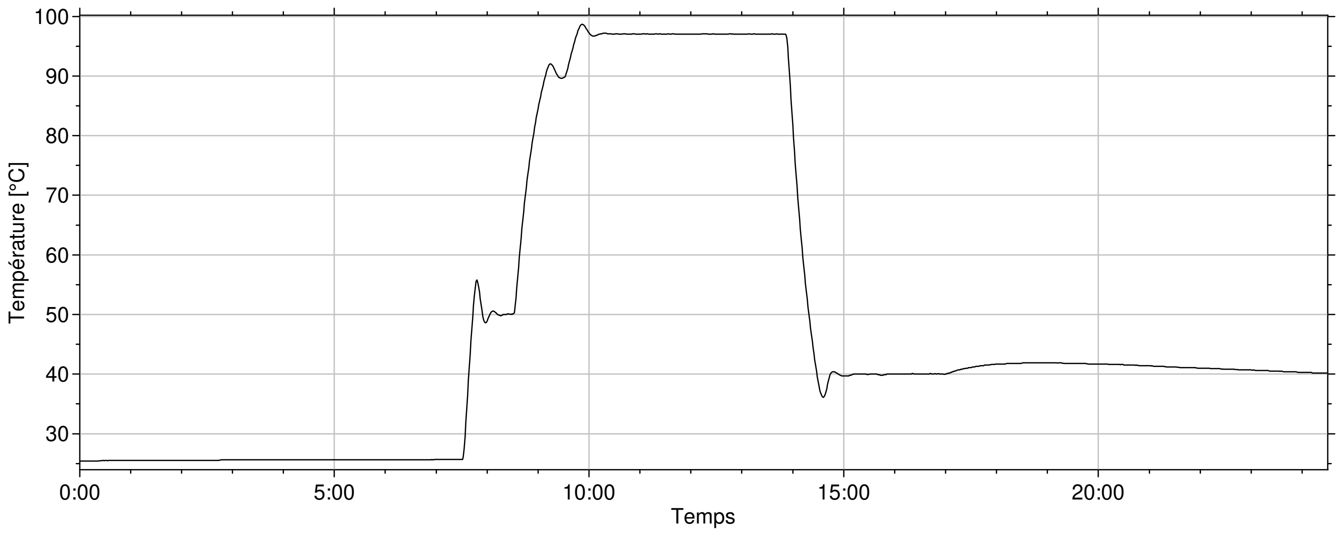

Gaia–Puissance du four

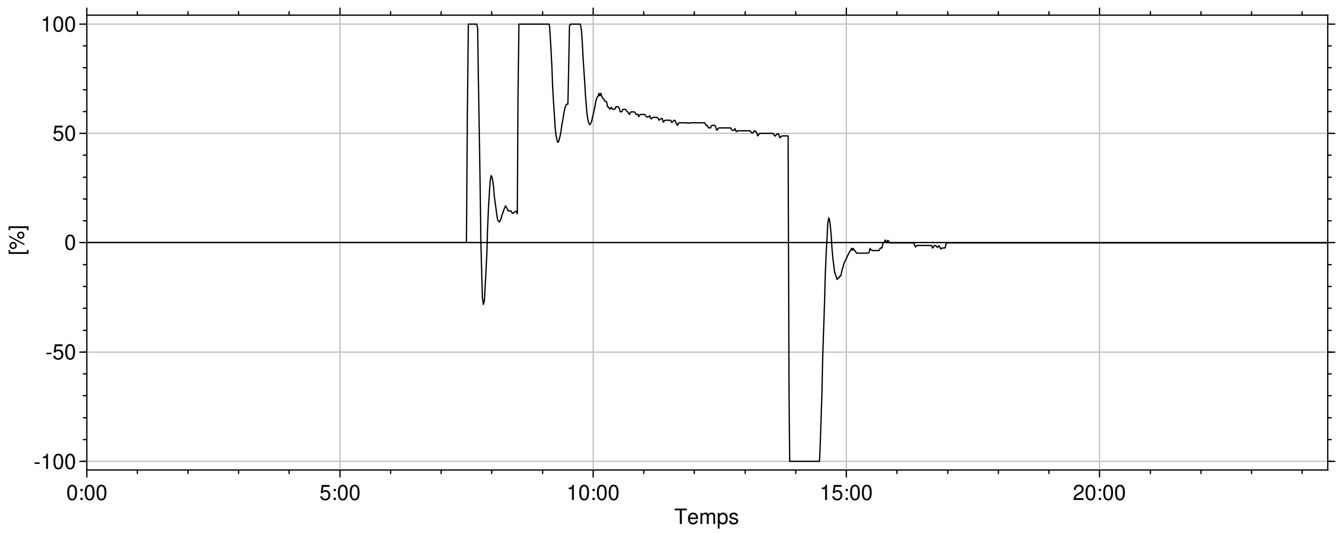

Gaia–Pression

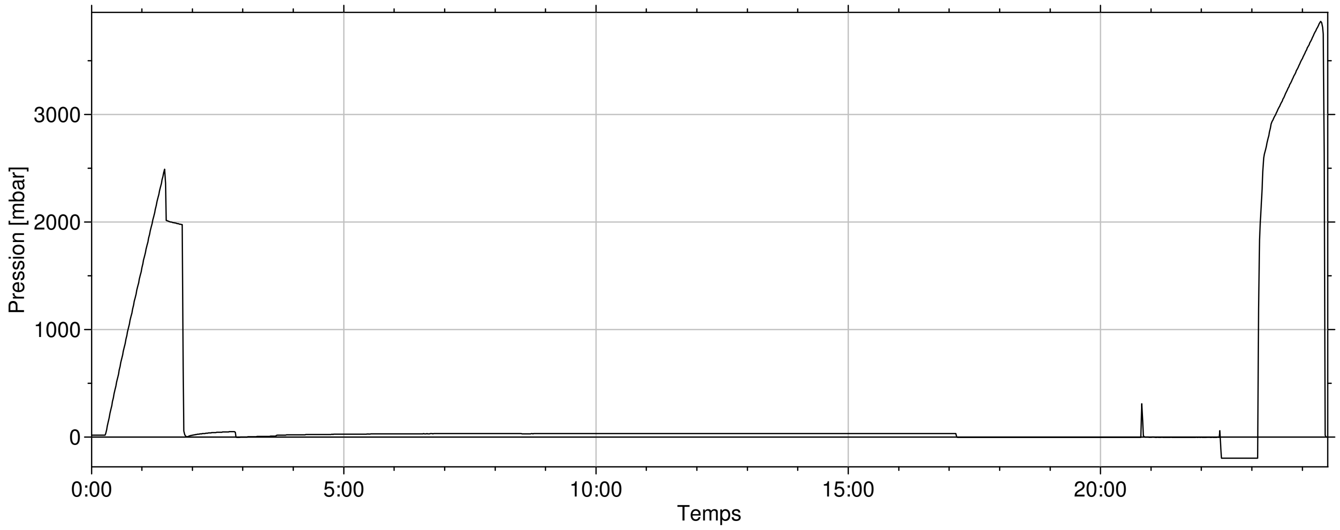

Gaia–Détecteur de flux

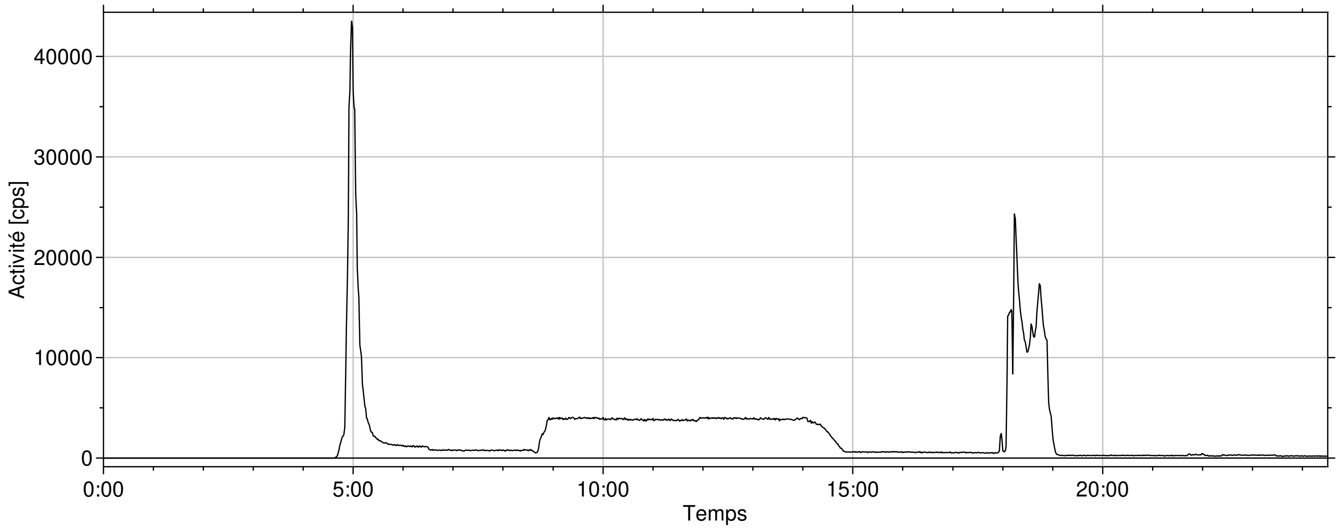

Gaia–Détecteur du réacteur

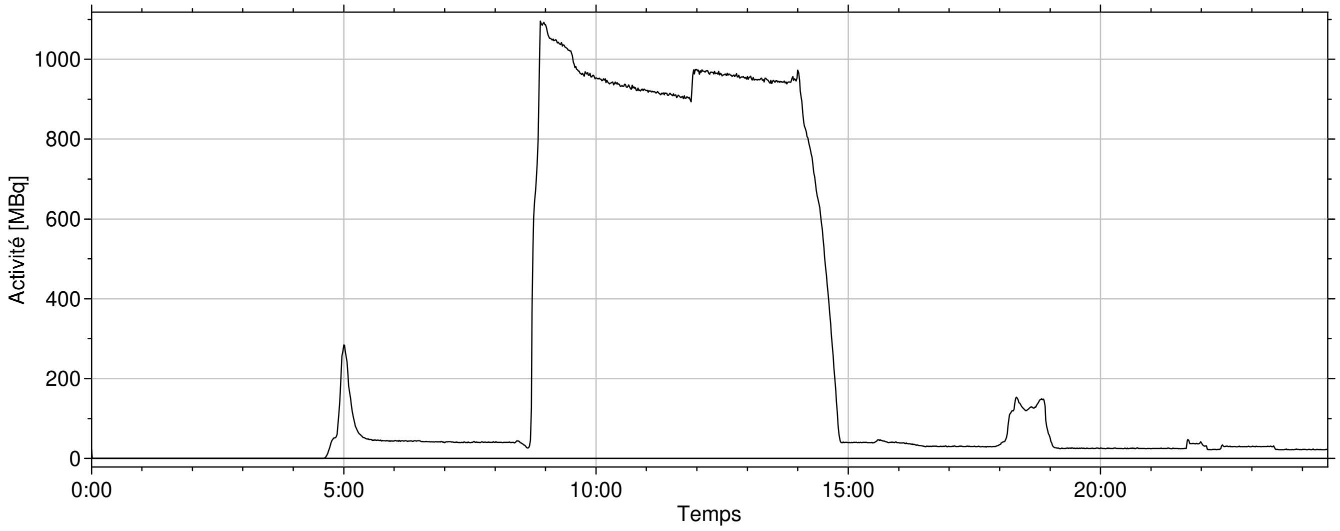

Gaia–Détecteur de la rampe 1

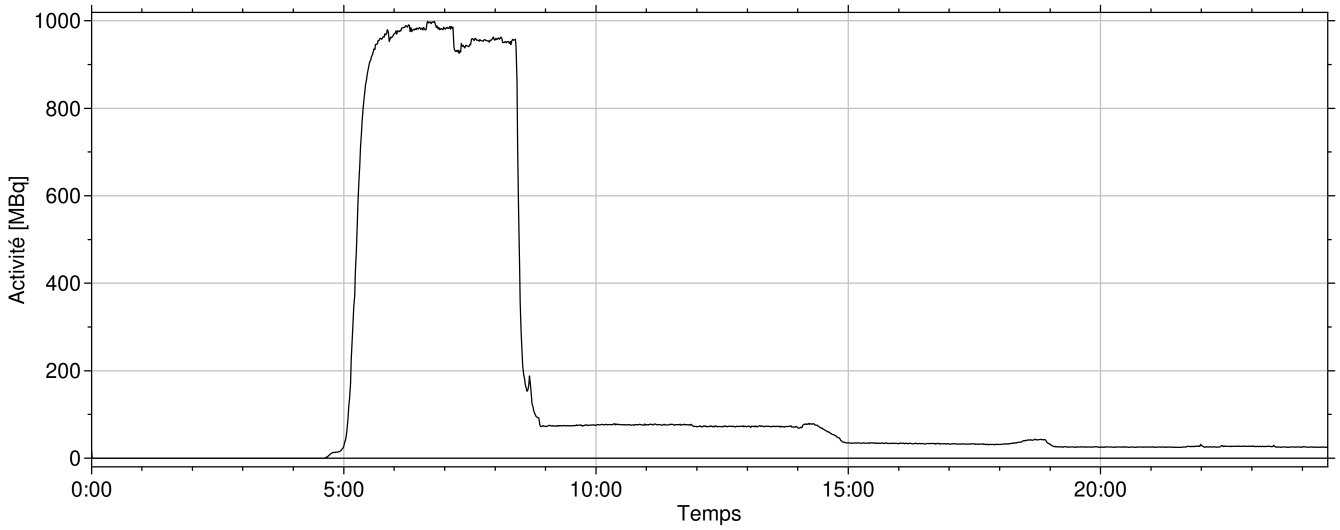

Gaia–Détecteur de la rampe 2

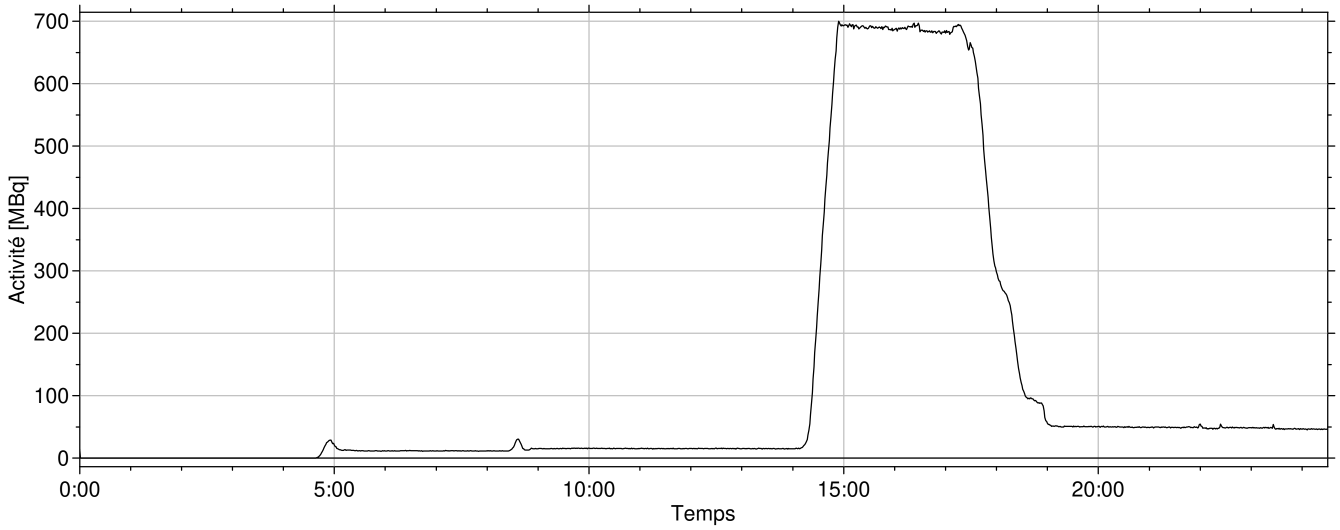

Luna–Position releveur

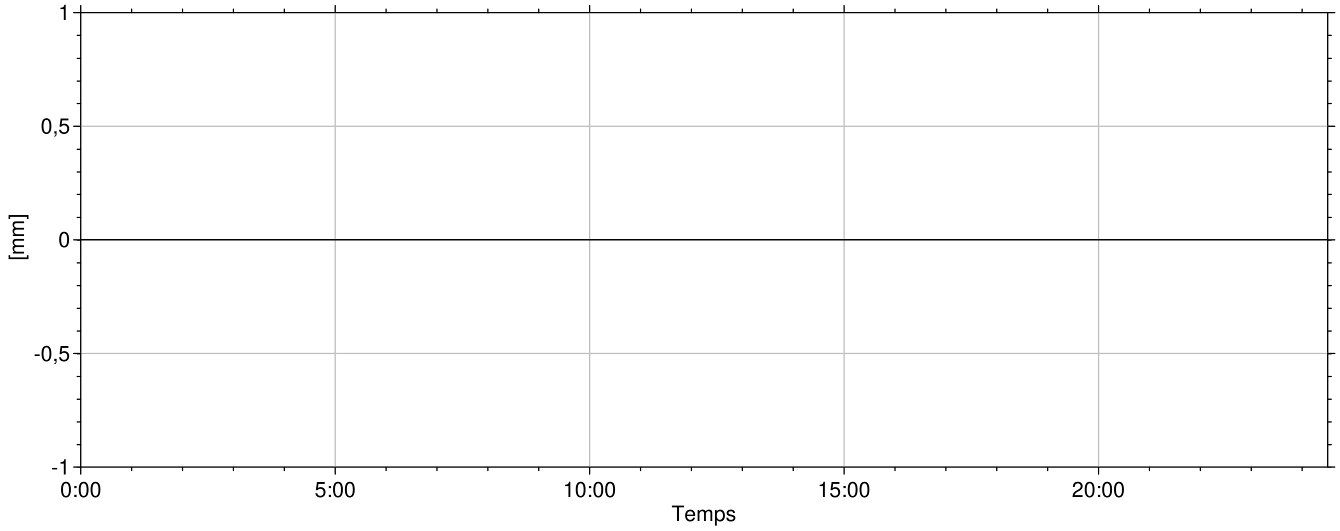

Luna–Température du four

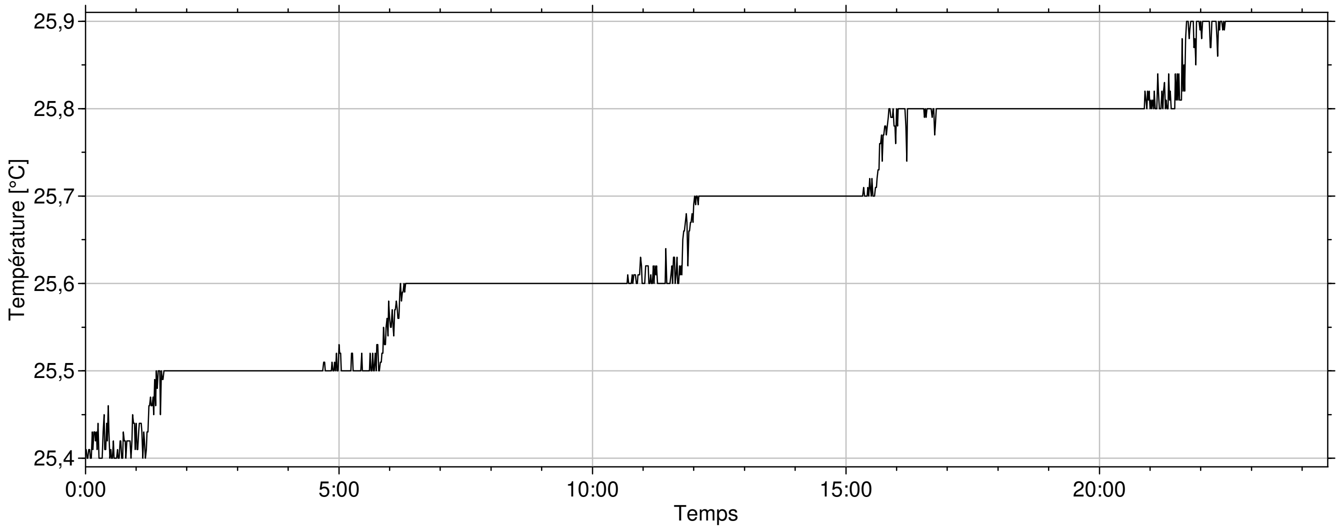

Luna–Puissance du four

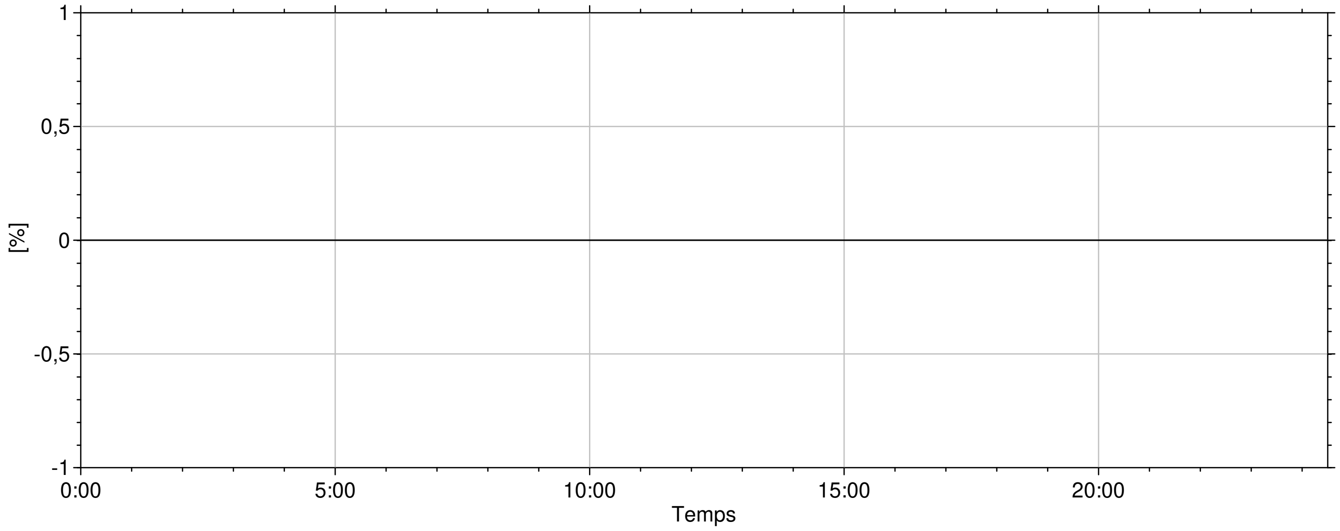

Luna–Détecteur du réacteur

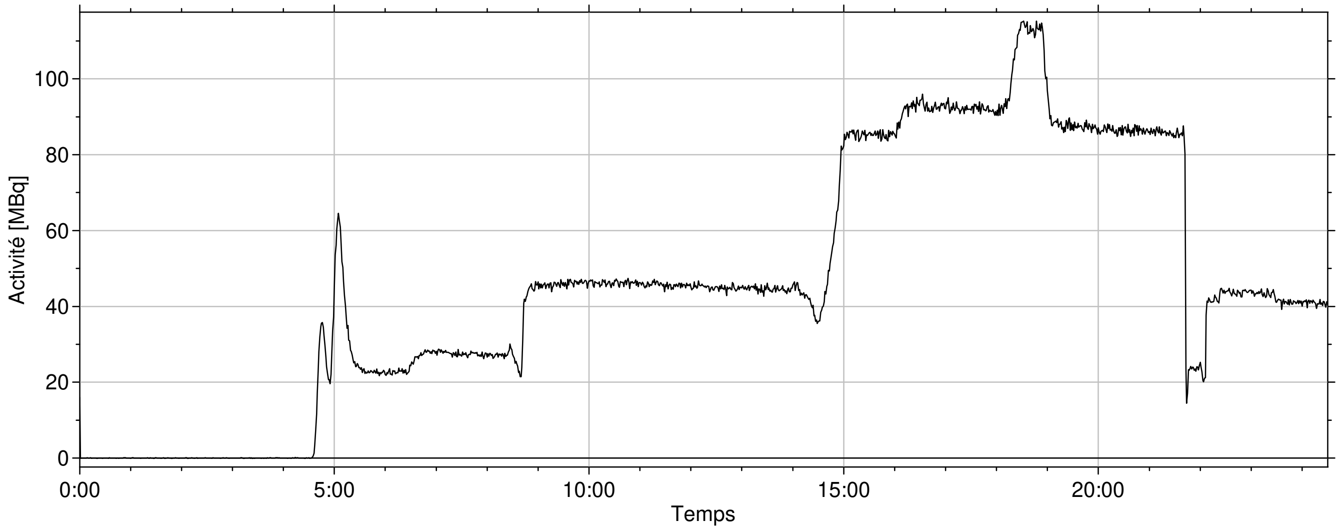

Luna–Tension du détecteur

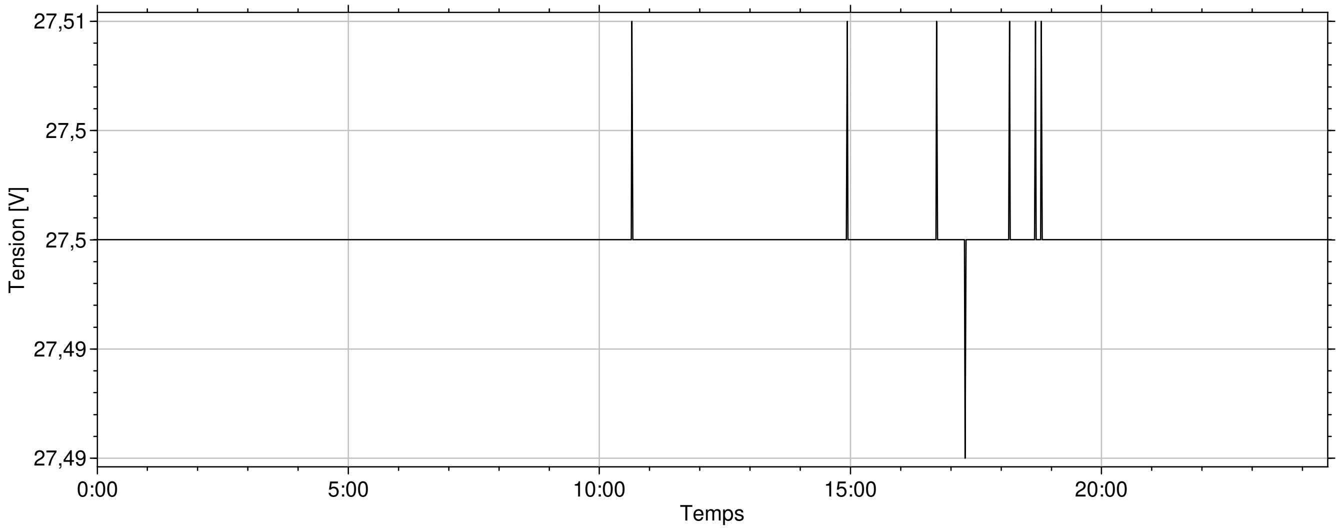

Supplement: Supplementary file 1 — Additional file 1. GMP-conditions synthesis report N°1. This document shows all details dealing with technique parameters of the Gaia/Luna Elysia-Raytest module during the first GMP-condition synthesis. [file 41181_2023_187_MOESM1_ESM.pdf]
