# Supplementary material for: Automated synthesis and quality control of [68Ga]Ga-PentixaFor using the Gaia/Luna Elysia-Raytest module for CXCR4 PET imaging
Source: EJNMMI Radiopharm Chem. 2023 Feb 7;8:4. doi: 10.1186/s41181-023-00187-2 (PMC9905377; doi:10.1186/s41181-023-00187-2)

No lot:	PTX GMP-2
Méthode:	Pentixafor Rev. 71 (01/09/2022 09:21:19)
No kit:	RT-01-H RT-H-220412001-1 1 et RT-101 RT-101-220510002-1 1
No peptide:	9916.0000.050/005 PenFor-05-20110901.01 1
Logiciel :	Gaia Control Rev. 2.2
Système:	Gaia: raytest Gaia, Numéro de série 35701, Rev. 1.30<br>Luna: raytest Luna, Numéro de série 35704, Rev. 1.04
Opérateur:	DANIEL (DANIEL)
Code kit:	Utilisé en R&D ou pour évaluation. Non valide en utilisation GMP!
Temps départ:	13/09/2022 09:22:44
Temps fin:	13/09/2022 09:47:06
Statut:	Synthèse complète

Préparation POS

✓ Mettre des gants stériles
✓ Resserer tous les raccords du kit fluidique
✓ Préparer le cartouche C18 SEPACK avec 5 ml d'éthanol absolu dans le petit becher
✓ Fixer la rampe A et installer la cartouche SCX entre A2 et B1 terminal
✓ Fixer la rampe B et installer la cartouche C18 entre B5 terminal et C2
✓ Fixer la rampe C
✓ Mettre le flacon de réaction sur le kit dans le four du GAIA
✓ Brancher la tubulure VYGON en A5 et la connecter au filtre 0,22 µm spécifique
✓ Connecter un aiguille STERICAN au filtre et l'inserer dans le flacon final de réaction
✓ Inserer une prise d'air 0,22 µm dans le flacon final de réaction
✓ Déconnecter le filtre 1,2 µm de la prise d'air B braun et connecter le filtre en A4
✓ Connecter la tubulure en A1 à l'embout de pression
✓ Positionner l'aiguille sterican jaune sur la tubulure en A1 terminal
✓ Inserer cette aiguille dans le flacon de dechet et inserer un prise d'air
✓ Faire des aller/retour avec le piston de la seringue d'éluant
✓ Connecter la seringue d'éluant C1 et la positionner sur la potence à droite
✓ Connecter la tubulure en C4 au flacon d'eau ppi et le positionner sur le support à droite
✓ Insérer la tubulure en C5 terminal dans la pompe perilstatique et le détécteur
✓ Connecter la tubulure en C5 à la sortie du générateur
✓ Connecter le flacon de NaCl 0,9 % (bleu) en B4 bleu
✓ Connecter le flacon d' ethanol 60 % (or) en B5 or
✓ Prélever 2,2mL de tampon à l'aide d'une seringue de 5mL et d'une aiguille micropine
✓ Connecter une aiguille sterican à cette seringue et prelever la totalité du tampon
✓ Ajuster la seringue et la connecter en B3
✓ Ouvrir les robinets de la poche de HCl 0,1 M dans le sens de l'élution

Résultats

Etape 25:	SCX Elution: Preparation:	902 MBq
Etape 29:	Labeling / Measuring Reactor Activity:	929 MBq
Etape 39:	C18: Purge / Measuring Empty Reaktor:	34 MBq
Etape 40:	C18: Purge / Measuring C18:	748 MBq
Etape 49:	Formulation: Purge / Measuring C18 Post_Elution:	41 MBq
Etape 50:	Venting Preparation / Measuring SCX Post_Elution:	32 MBq
Etape 59:	Filter Integrity Test: Measuring Bubble Point Value:	3826 mbar

Rendement:	78%
Durée de la synthèse:	0:09:49 (589s)

Journal de la synthèse

13/09/2022 09:22:44	Synthèse démarrée
+00:15	Etape 1 terminée: C18 Purge
+01:33	Etape 2 terminée: Kit Integrity Test: Pressurizing

+01:38	Etape 3 terminée: Kit Integrity Test: Equilibration
+01:53	Etape 4 terminée: Kit Integrity Test: Measuring
+01:58	Etape 5 terminée: Kit Integrity Test: Venting
+02:08	Etape 6 terminée: NaCl Syringe Preparation
+02:23	Etape 7 terminée: Buffer Addition: Preparation
+02:25	Etape 8 terminée: Buffer Addition: Preparation Venting
+02:55	Etape 9 terminée: Buffer Addition
+02:59	Etape 10 terminée: Buffer Addition: Venting
+03:29	Etape 11 terminée: Cartridge Activation: SCX + C18
+03:39	Etape 12 terminée: Cartridge Activation: C18
+03:44	Etape 13 terminée: Cartridge Activation: Purge Preparation
+03:49	Etape 14 terminée: Cartridge Activation: Purge 1
+03:59	Etape 15 terminée: Cartridge Activation: Purge 2
+04:09	Etape 16 terminée: Cartridge Activation: Purge 3
+04:13	Etape 17 terminée: Cartridge Activation: Venting
+06:23	Etape 18 terminée: Generator Elution: Collect
+06:33	Etape 19 terminée: Generator Elution: Waste
+06:43	Etape 20 terminée: SCX Washing: Line
+06:53	Etape 21 terminée: SCX Washing
+06:58	Etape 22 terminée: SCX Washing: Line Purge Preparation
+07:13	Etape 23 terminée: SCX Washing: Line Purge
+07:24	Etape 24 terminée: SCX Washing: Cartridge Purge
+07:36	Etape 25 terminée: SCX Elution: Preparation
+08:36	Etape 26 terminée: SCX Elution 1
+09:36	Etape 27 terminée: SCX Elution 2
+09:51	Etape 28 terminée: SCX Elution: Purge
+09:56	Etape 29 terminée: Labeling / Measuring Reactor Activity
+11:56	Etape 30 terminée: Labeling Part 1
+12:06	Etape 31 terminée: Labeling / Lift Purge
+13:57	Etape 32 terminée: Labeling Part 2
+14:09	Etape 33 terminée: Delution
+14:19	Etape 34 terminée: C18 Peptide Trapping: Preparation
+15:34	Etape 35 terminée: C18 Peptide Trapping
+15:52	Etape 36 terminée: Washing Reaktor
+16:02	Etape 37 terminée: C18 Washing: Preparation
+17:03	Etape 38 terminée: C18 Washing
+17:08	Etape 39 terminée: C18: Purge / Measuring Empty Reaktor
+17:13	Etape 40 terminée: C18: Purge / Measuring C18
+17:23	Etape 41 terminée: C18: Elution: EtOH
+17:33	Etape 42 terminée: C18: Elution: Water
+17:43	Etape 43 terminée: C18: Elution: EtOH
+17:58	Etape 44 terminée: C18: Elution: Water
+18:18	Etape 45 terminée: C18: Elution: EtOH
+18:38	Etape 46 terminée: C18: Elution: Water
+18:58	Etape 47 terminée: C18: Elution: EtOH
+20:28	Etape 48 terminée: Formulation
+20:48	Etape 49 terminée: Formulation: Purge / Measuring C18 Post_Elution
+20:54	Etape 50 terminée: Venting Preparation / Measuring SCX Post_Elution
+21:39	Etape 51 terminée: Remove Product, Connect Waste
+22:09	Etape 52 terminée: Filter Integrity Test: Filter Purge
+22:14	Etape 53 terminée: Filter Integrity Test: Line Purge Preparation
+22:29	Etape 54 terminée: Filter Integrity Test: Line Purge 1
+22:45	Etape 55 terminée: Filter Integrity Test: Line Purge 2
+22:55	Etape 56 terminée: Filter Integrity Test: Line Purge 3
+23:02	Etape 57 terminée: Filter Integrity Test: Pressurizing
+23:12	Etape 58 terminée: Filter Integrity Test: Approaching Bubble Point Value
+24:17	Etape 59 terminée: Filter Integrity Test: Measuring Bubble Point Value
+24:22	Etape 60 terminée: Venting
+24:22	Synthèse complète: 13/09/2022 09:47:06

Gaia–Vitesse de la pompe

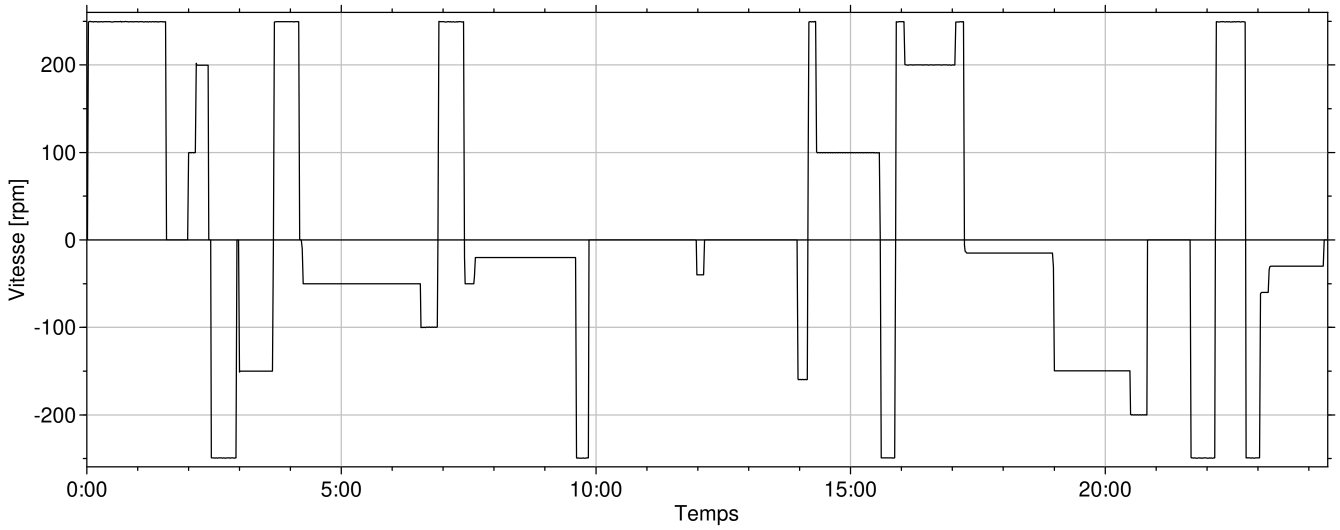

Gaia–Température du four

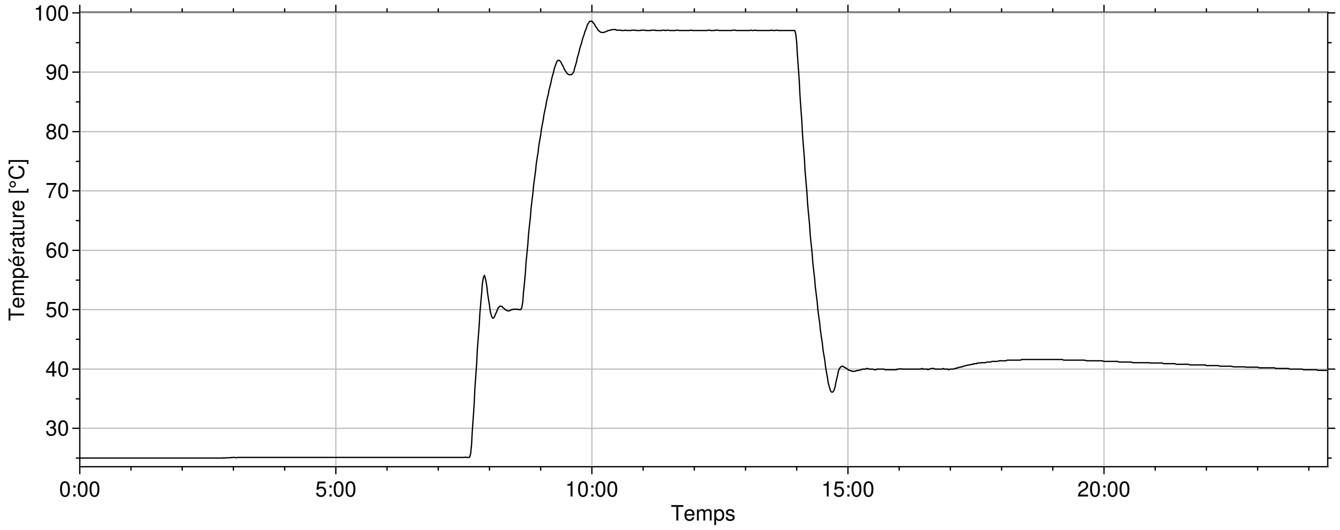

Gaia–Puissance du four

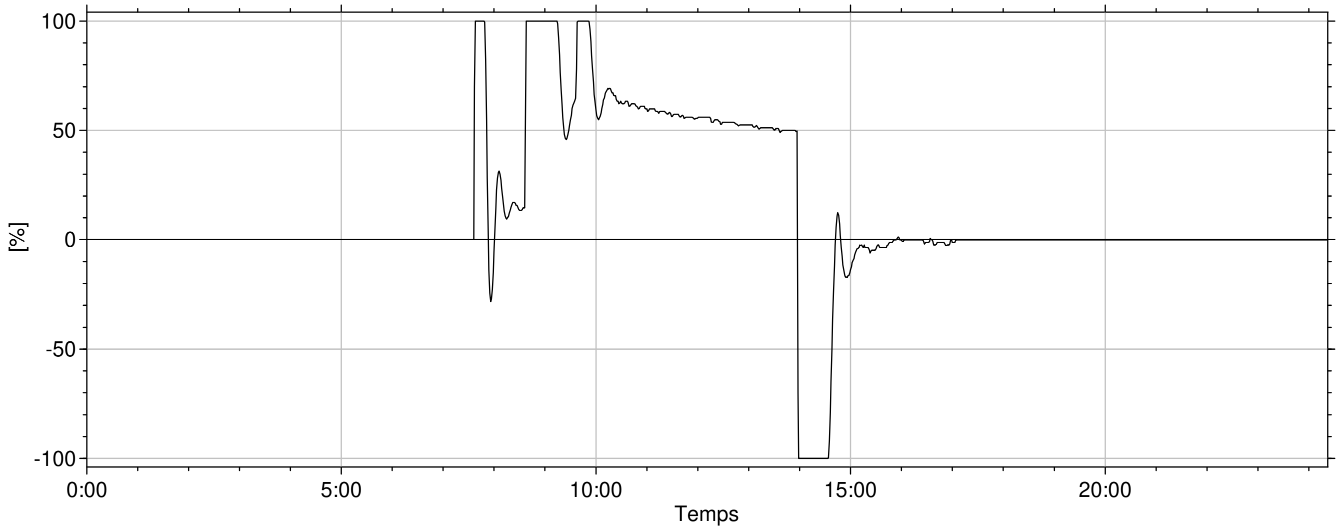

Gaia–Pression

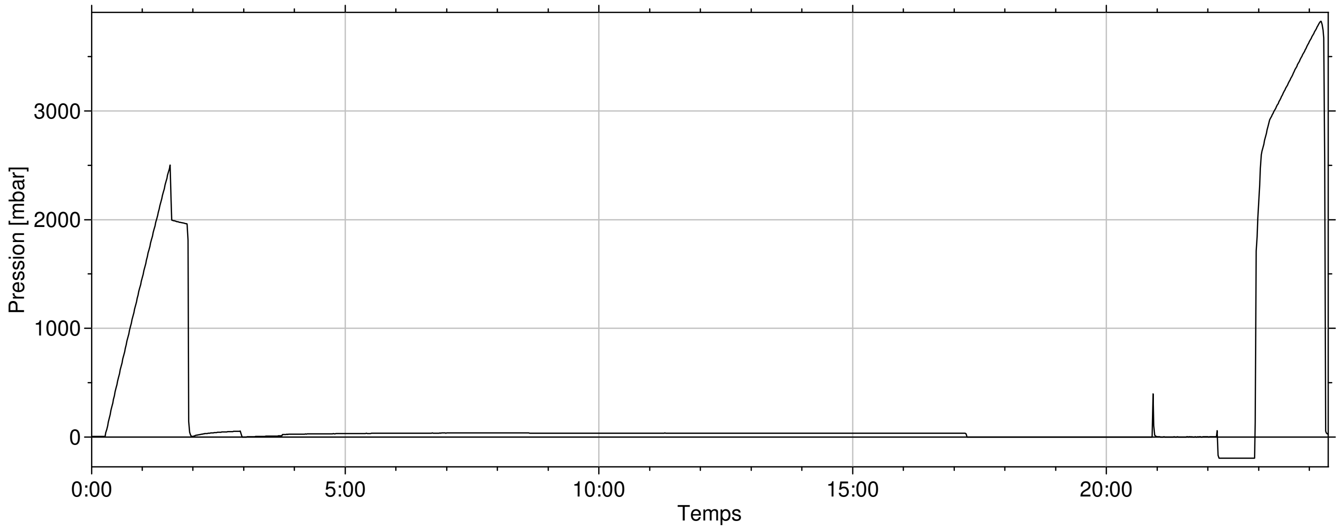

Gaia–Détecteur de flux

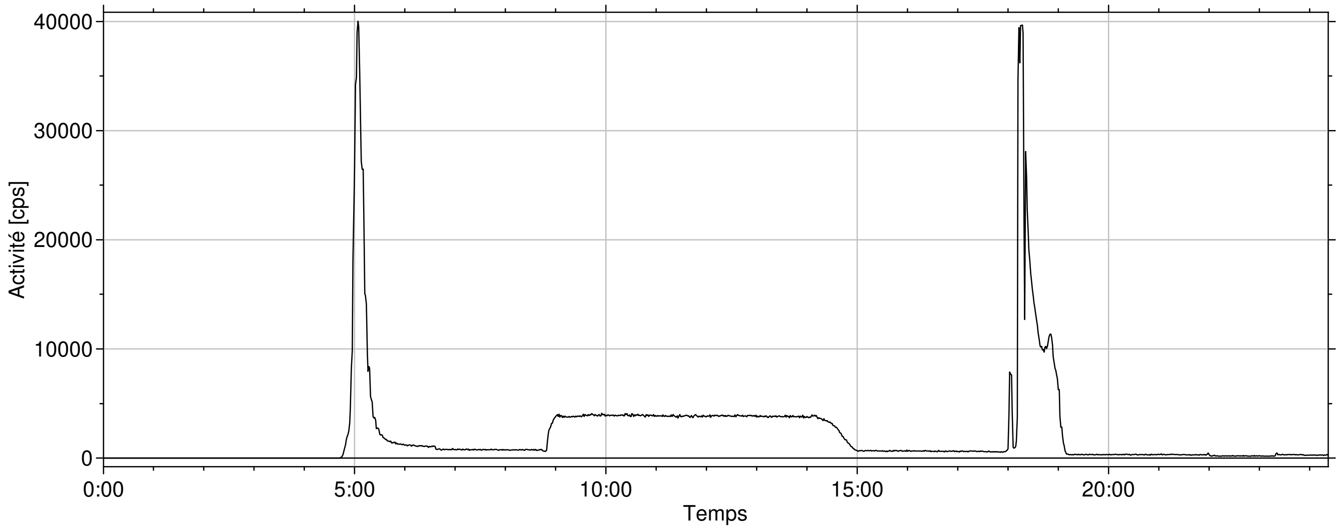

Gaia–Détecteur du réacteur

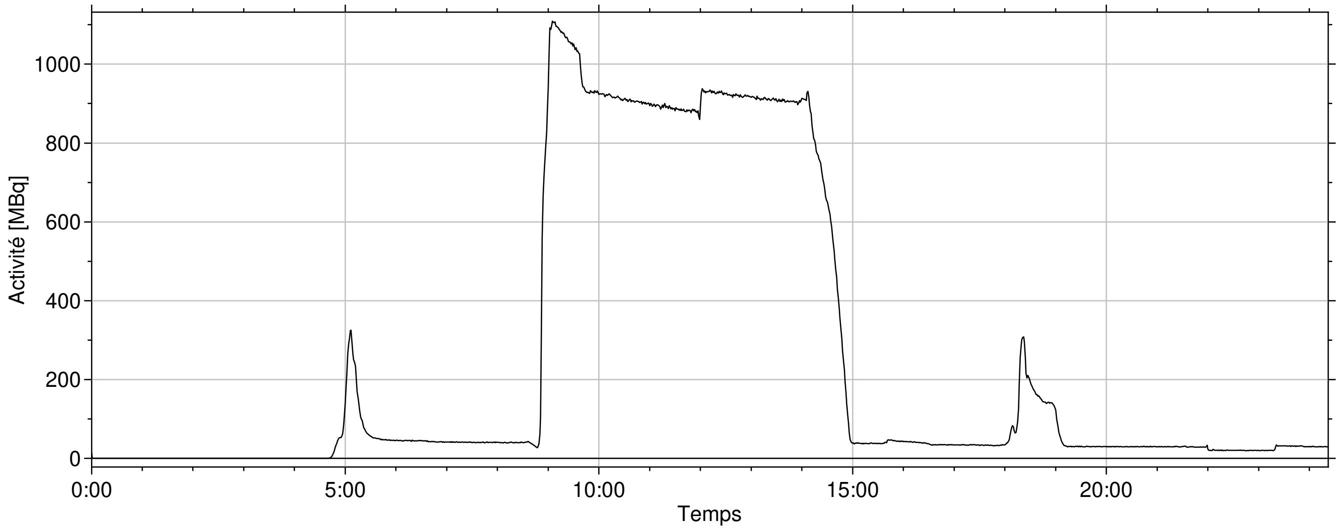

Gaia–Détecteur de la rampe 1

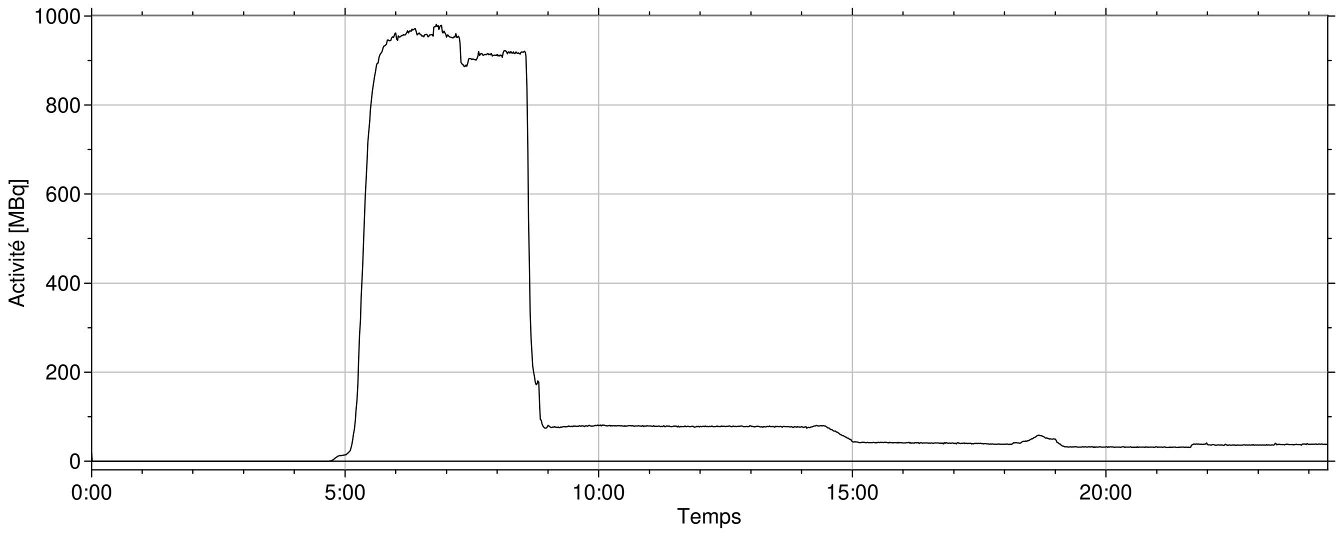

Gaia–Détecteur de la rampe 2

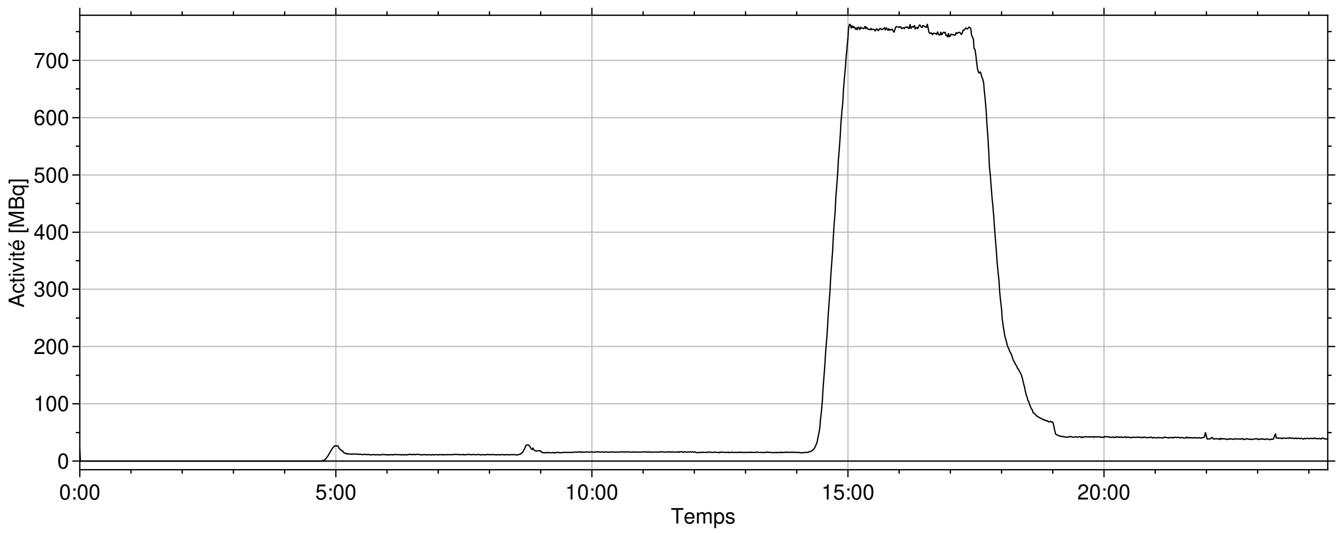

Luna–Position releveur

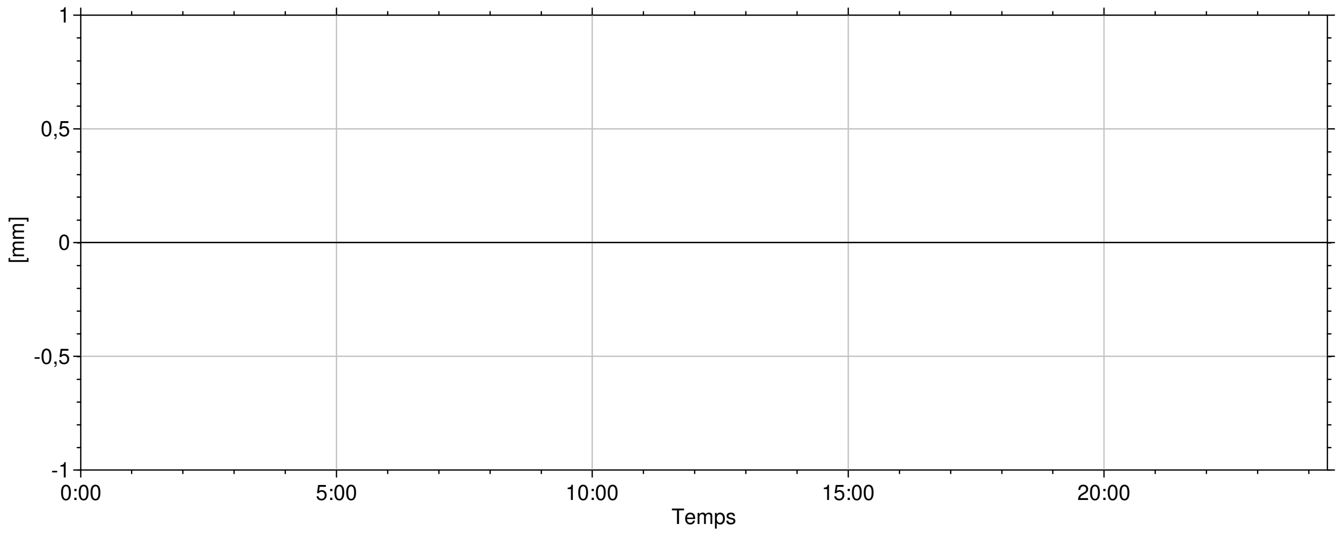

Luna–Température du four

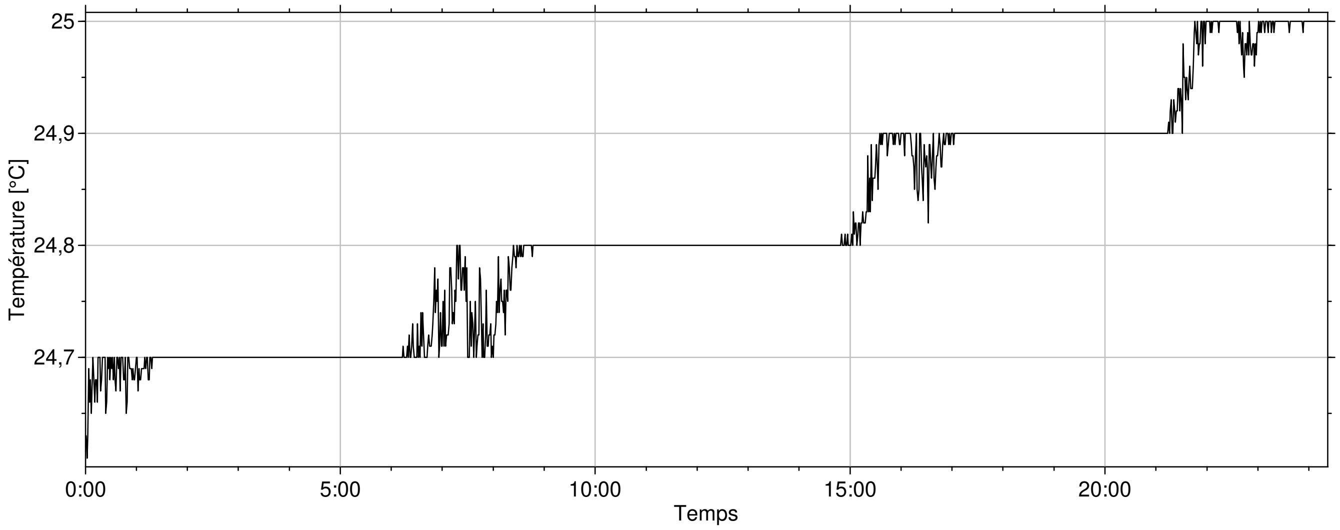

Luna–Puissance du four

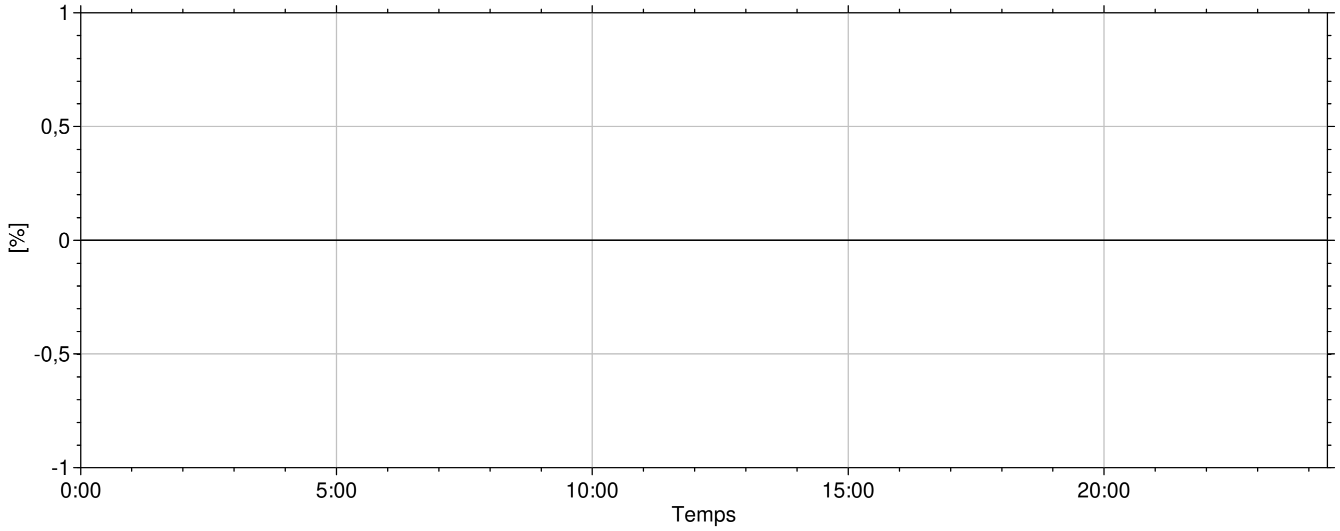

Luna–Détecteur du réacteur

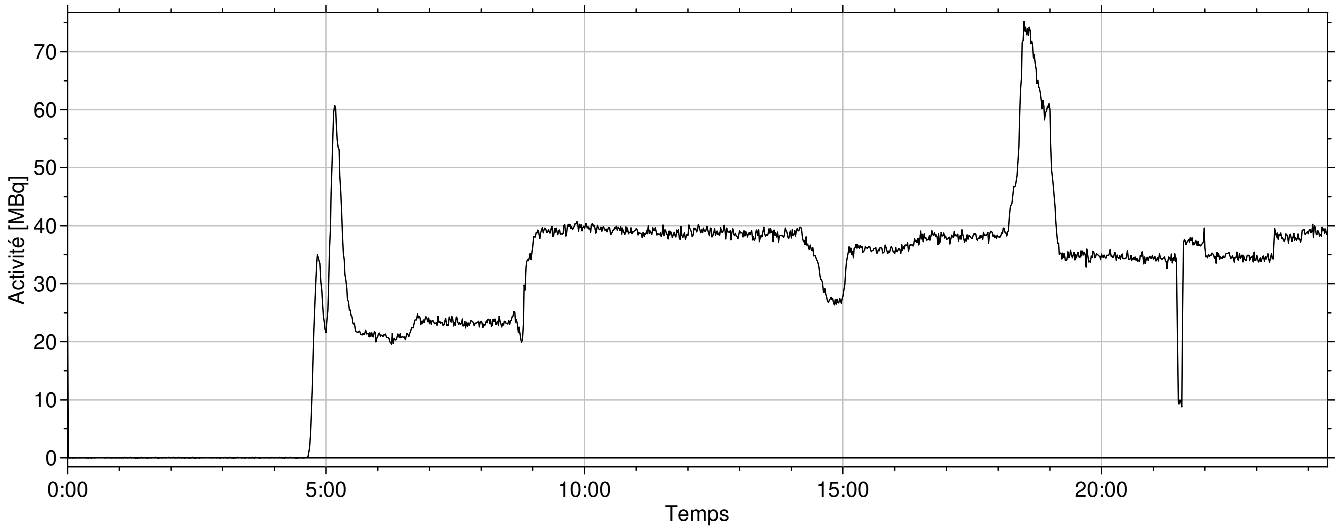

Luna–Tension du détecteur

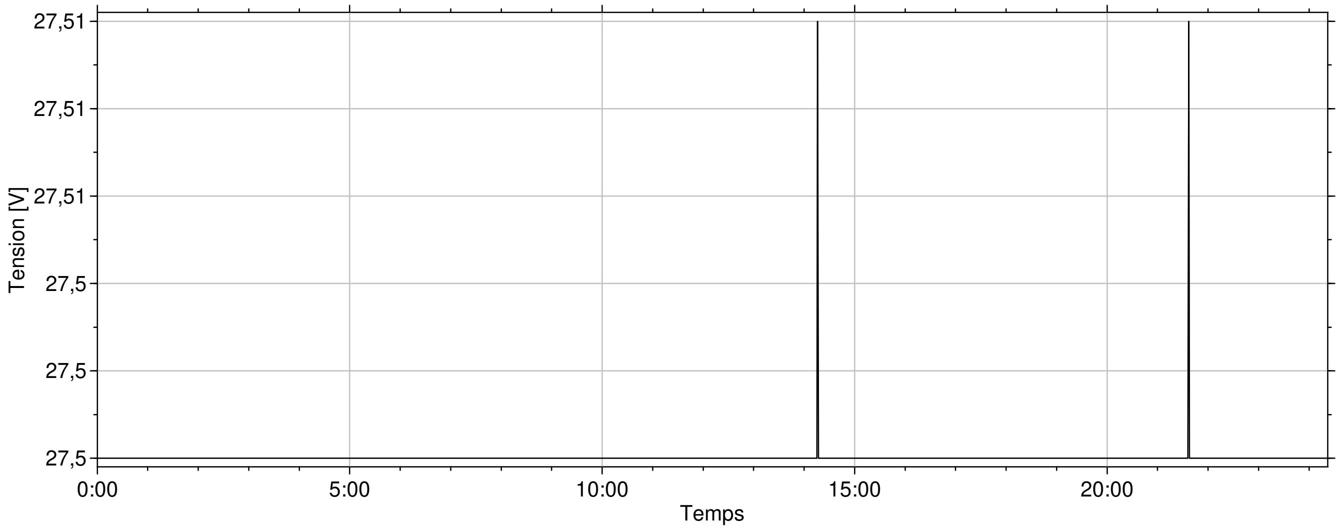

Supplement: Supplementary file 2 — Additional file 2. GMP-conditions synthesis report N°2. This document shows all details dealing with technique parameters of the Gaia/Luna Elysia-Raytest module during the second GMP-condition synthesis. [file 41181_2023_187_MOESM2_ESM.pdf]
