# Supplementary material for: Automated synthesis and quality control of [68Ga]Ga-PentixaFor using the Gaia/Luna Elysia-Raytest module for CXCR4 PET imaging
Source: EJNMMI Radiopharm Chem. 2023 Feb 7;8:4. doi: 10.1186/s41181-023-00187-2 (PMC9905377; doi:10.1186/s41181-023-00187-2)

No lot:	PTX GMP 3
Méthode:	Pentixafor Rev. 76 (20/09/2022 08:25:52)
No kit:	RT-101 RT-101-220510002-1 1RT-01-H RT-H-220412001-1 1
No peptide:	9916.0000.050/005 PenFor-05-20110901.01 1
Logiciel :	Gaia Control Rev. 2.2
Système:	Gaia: raytest Gaia, Numéro de série 35701, Rev. 1.30<br>Luna: raytest Luna, Numéro de série 35704, Rev. 1.04
Opérateur:	mouraeff (mouraeff)
Code kit:	Utilisé en R&D ou pour évaluation. Non valide en utilisation GMP!
Temps départ:	20/09/2022 09:56:04
Temps fin:	20/09/2022 10:20:49
Statut:	Synthèse complète

Préparation POS

✓ Mettre des gants stériles
✓ Resserer tous les raccords du kit fluidique
✓ Préparer le cartouche C18 SEPACK avec 5 ml d'éthanol absolu dans le petit becher
✓ Fixer la rampe A et installer la cartouche SCX entre A2 et B1 terminal
✓ Fixer la rampe B et installer la cartouche C18 entre B5 terminal et C2
✓ Fixer la rampe C
✓ Mettre le flacon de réaction sur le kit dans le four du GAIA
✓ Brancher la tubulure VYGON en A5 et la connecter au filtre 0,22 µm spécifique
✓ Connecter un aiguille STERICAN au filtre et l'inserer dans le flacon final de réaction
✓ Inserer une prise d'air 0,22 µm dans le flacon final de réaction
✓ Déconnecter le filtre 1,2 µm de la prise d’air B braun et connecter le filtre en A4
✓ Connecter la tubulure en A1 à l’embout de pression
✓ Positionner l’aiguille sterican jaune sur la tubulure en A1 terminal
✓ Inserer cette aiguille dans le flacon de dechet et inserer un prise d'air
✓ Faire des aller/retour avec le piston de la seringue d’éluant
✓ Connecter la seringue d’éluant C1 et la positionner sur la potence à droite
✓ Connecter la tubulure en C4 au flacon d’eau ppi et le positionner sur le support à droite
✓ Insérer la tubulure en C5 terminal dans la pompe perilstatique et le détécteur
✓ Connecter la tubulure en C5 à la sortie du générateur
✓ Connecter le flacon de NaCl 0,9 % (bleu) en B4 bleu
✓ Connecter le flacon d' ethanol 60 % (or) en B5 or
✓ Prélever 2,2mL de tampon à l’aide d’une seringue de 5mL et d’une aiguille micropine
✓ Connecter une aiguille sterican à cette seringue et prelever la totalité du tampon
✓ Ajuster la seringue et la connecter en B3
✓ Ouvrir les robinets de la poche de HCl 0,1 M dans le sens de l'élution

Résultats

Etape 25:	SCX Elution: Preparation:	892 MBq
Etape 29:	Labeling / Measuring Reactor Activity:	931 MBq
Etape 39:	C18: Purge / Measuring Empty Reaktor:	21 MBq
Etape 40:	C18: Purge / Measuring C18:	697 MBq
Etape 49:	Formulation: Purge / Measuring C18 Post_Elution:	56 MBq
Etape 50:	Venting Preparation / Measuring SCX Post_Elution:	13 MBq
Etape 59:	Filter Integrity Test: Measuring Bubble Point Value:	3899 mbar

Rendement:	72%
Durée de la synthèse:	0:09:49 (589s)

Journal de la synthèse

20/09/2022 09:56:04	Synthèse démarrée
+00:16	Etape 1 terminée: C18 Purge
+01:34	Etape 2 terminée: Kit Integrity Test: Pressurizing

+01:39	Etape 3 terminée: Kit Integrity Test: Equilibration
+01:54	Etape 4 terminée: Kit Integrity Test: Measuring
+02:00	Etape 5 terminée: Kit Integrity Test: Venting
+02:10	Etape 6 terminée: NaCl Syringe Preparation
+02:25	Etape 7 terminée: Buffer Addition: Preparation
+02:27	Etape 8 terminée: Buffer Addition: Preparation Venting
+02:57	Etape 9 terminée: Buffer Addition
+03:00	Etape 10 terminée: Buffer Addition: Venting
+03:30	Etape 11 terminée: Cartridge Activation: SCX + C18
+03:40	Etape 12 terminée: Cartridge Activation: C18
+03:45	Etape 13 terminée: Cartridge Activation: Purge Preparation
+03:51	Etape 14 terminée: Cartridge Activation: Purge 1
+04:01	Etape 15 terminée: Cartridge Activation: Purge 2
+04:11	Etape 16 terminée: Cartridge Activation: Purge 3
+04:14	Etape 17 terminée: Cartridge Activation: Venting
+06:24	Etape 18 terminée: Generator Elution: Collect
+06:34	Etape 19 terminée: Generator Elution: Waste
+06:44	Etape 20 terminée: SCX Washing: Line
+06:54	Etape 21 terminée: SCX Washing
+06:59	Etape 22 terminée: SCX Washing: Line Purge Preparation
+07:15	Etape 23 terminée: SCX Washing: Line Purge
+07:25	Etape 24 terminée: SCX Washing: Cartridge Purge
+07:37	Etape 25 terminée: SCX Elution: Preparation
+08:37	Etape 26 terminée: SCX Elution 1
+09:37	Etape 27 terminée: SCX Elution 2
+09:52	Etape 28 terminée: SCX Elution: Purge
+09:57	Etape 29 terminée: Labeling / Measuring Reactor Activity
+11:57	Etape 30 terminée: Labeling Part 1
+12:08	Etape 31 terminée: Labeling / Lift Purge
+13:58	Etape 32 terminée: Labeling Part 2
+14:10	Etape 33 terminée: Delution
+14:20	Etape 34 terminée: C18 Peptide Trapping: Preparation
+15:35	Etape 35 terminée: C18 Peptide Trapping
+15:53	Etape 36 terminée: Washing Reaktor
+16:04	Etape 37 terminée: C18 Washing: Preparation
+17:04	Etape 38 terminée: C18 Washing
+17:09	Etape 39 terminée: C18: Purge / Measuring Empty Reaktor
+17:14	Etape 40 terminée: C18: Purge / Measuring C18
+17:24	Etape 41 terminée: C18: Elution: EtOH
+17:34	Etape 42 terminée: C18: Elution: Water
+17:44	Etape 43 terminée: C18: Elution: EtOH
+17:59	Etape 44 terminée: C18: Elution: Water
+18:19	Etape 45 terminée: C18: Elution: EtOH
+18:39	Etape 46 terminée: C18: Elution: Water
+19:00	Etape 47 terminée: C18: Elution: EtOH
+20:30	Etape 48 terminée: Formulation
+20:50	Etape 49 terminée: Formulation: Purge / Measuring C18 Post_Elution
+20:55	Etape 50 terminée: Venting Preparation / Measuring SCX Post_Elution
+21:05	Etape 51 terminée: Remove Product, Connect Waste
+21:35	Etape 52 terminée: Filter Integrity Test: Filter Purge
+21:40	Etape 53 terminée: Filter Integrity Test: Line Purge Preparation
+21:56	Etape 54 terminée: Filter Integrity Test: Line Purge 1
+22:11	Etape 55 terminée: Filter Integrity Test: Line Purge 2
+22:21	Etape 56 terminée: Filter Integrity Test: Line Purge 3
+22:35	Etape 57 terminée: Filter Integrity Test: Pressurizing
+22:48	Etape 58 terminée: Filter Integrity Test: Approaching Bubble Point Value
+24:40	Etape 59 terminée: Filter Integrity Test: Measuring Bubble Point Value
+24:45	Etape 60 terminée: Venting
+24:45	Synthèse complète: 20/09/2022 10:20:49

Gaia–Vitesse de la pompe

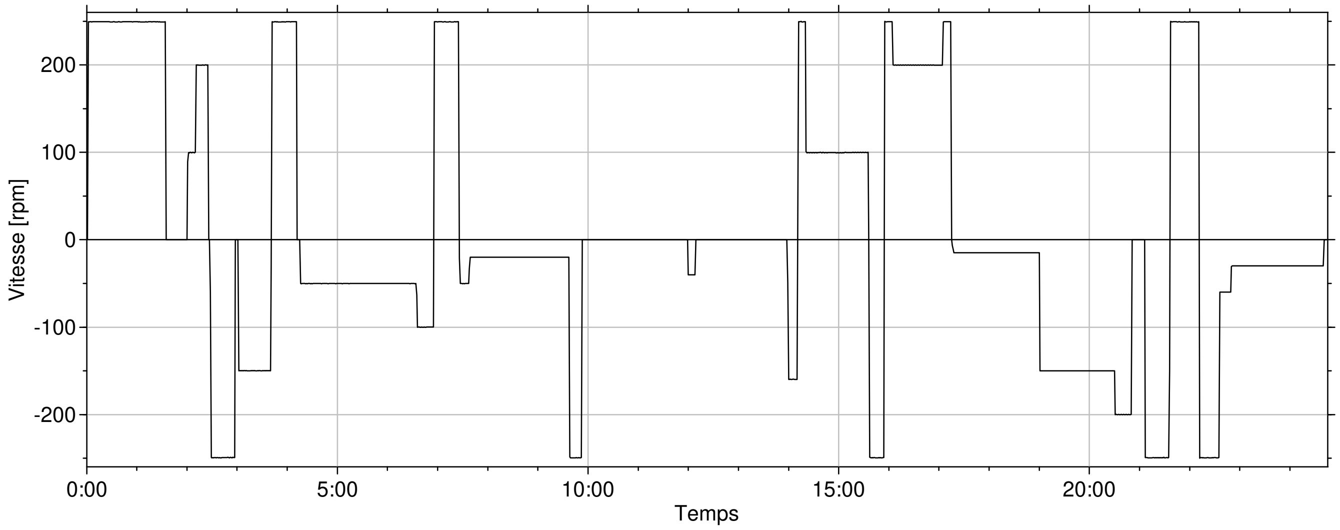

Gaia–Température du four

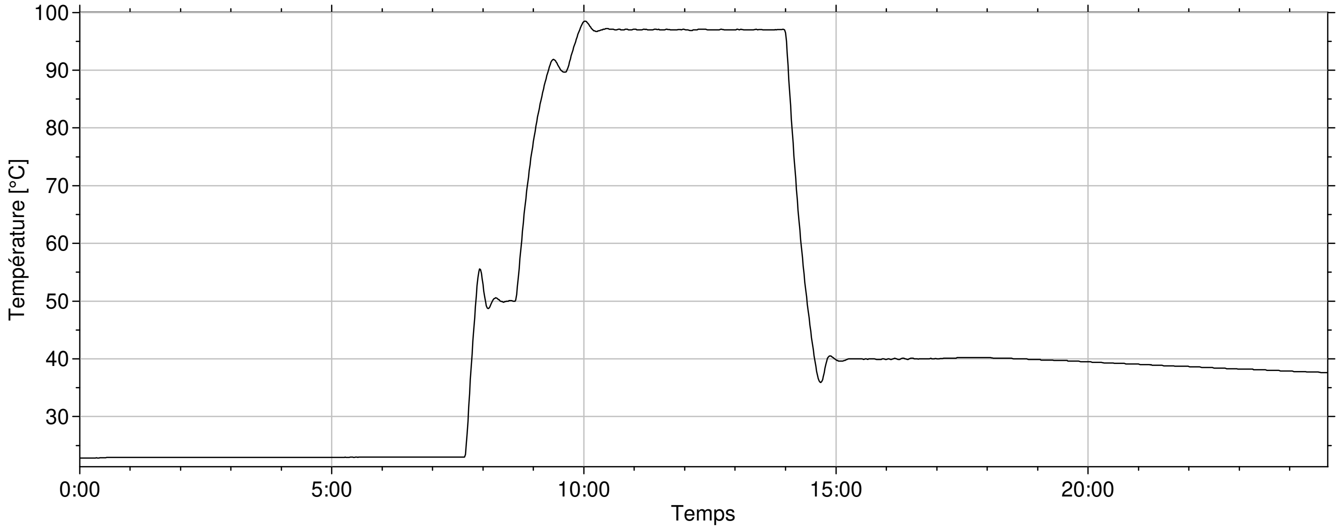

Gaia–Puissance du four

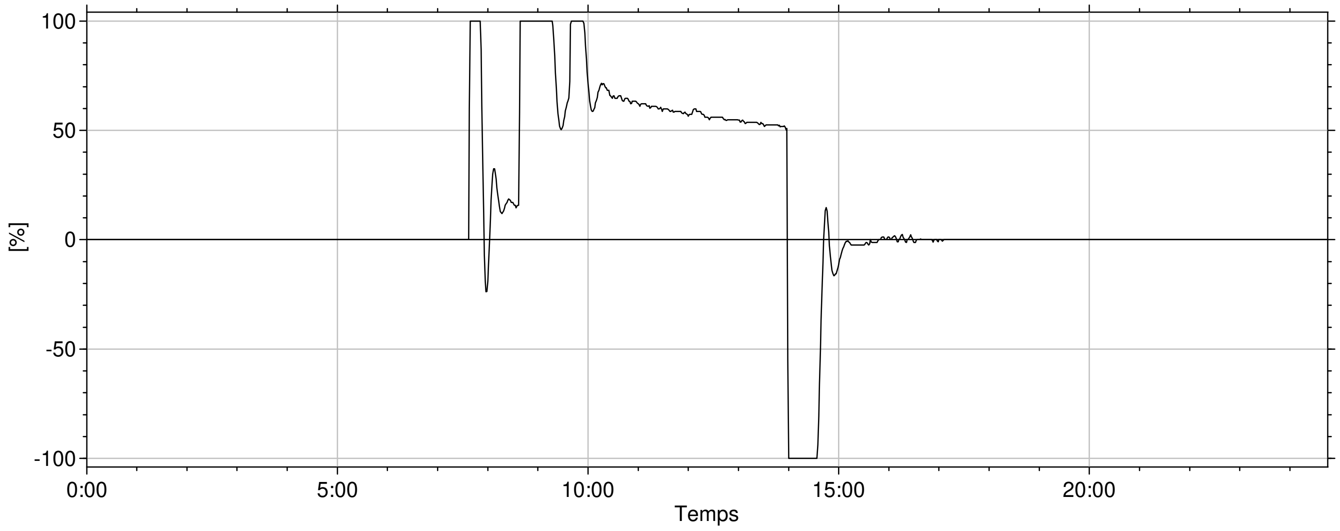

Gaia–Pression

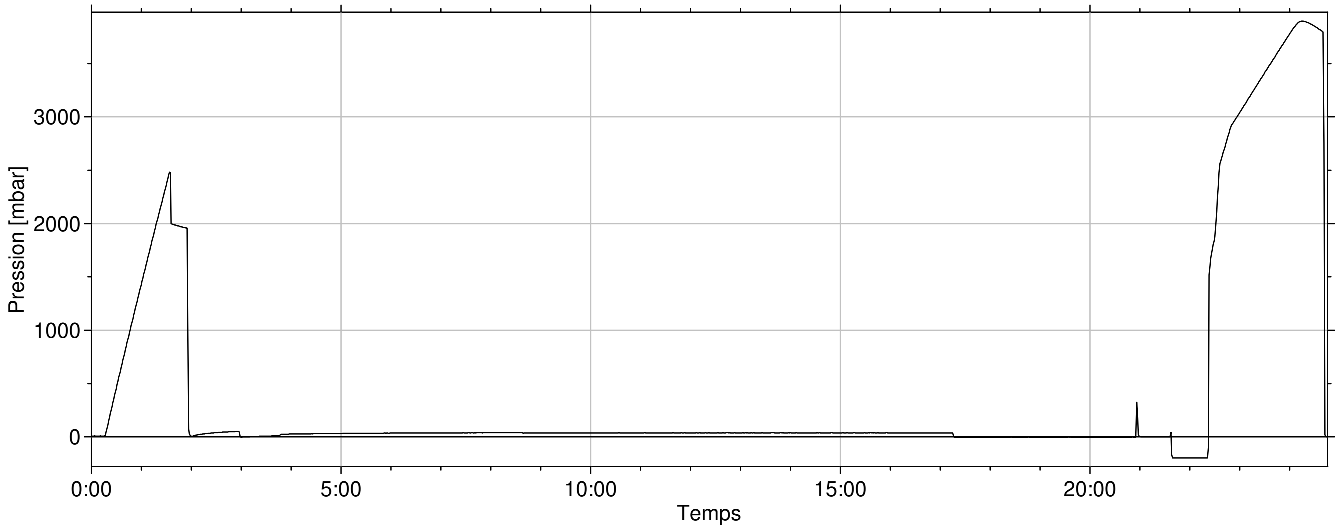

Gaia–Détecteur de flux

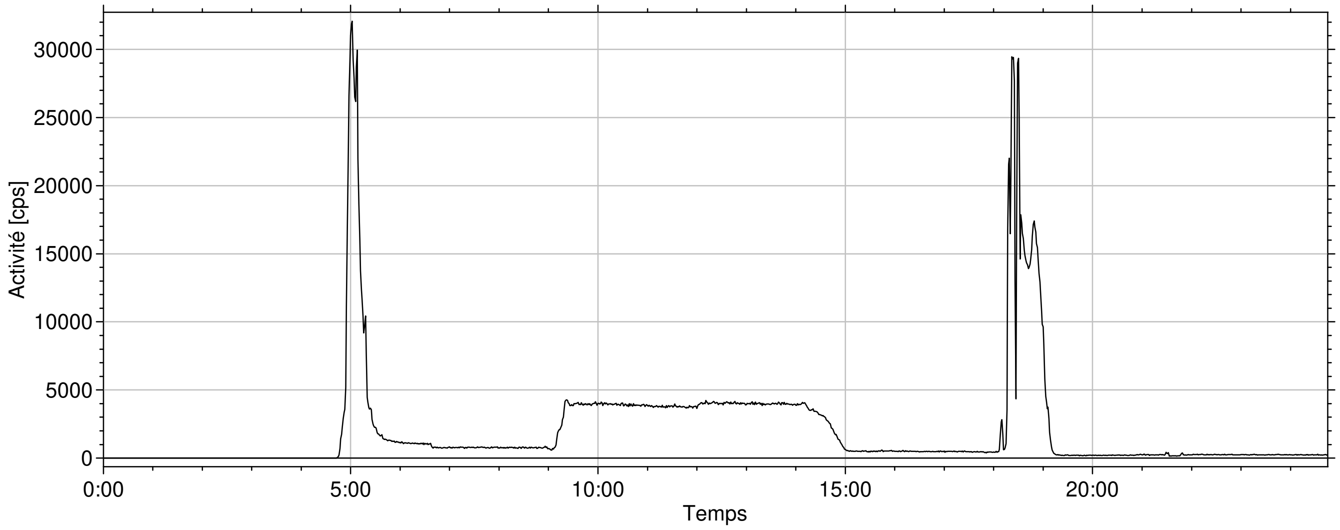

Gaia–Détecteur du réacteur

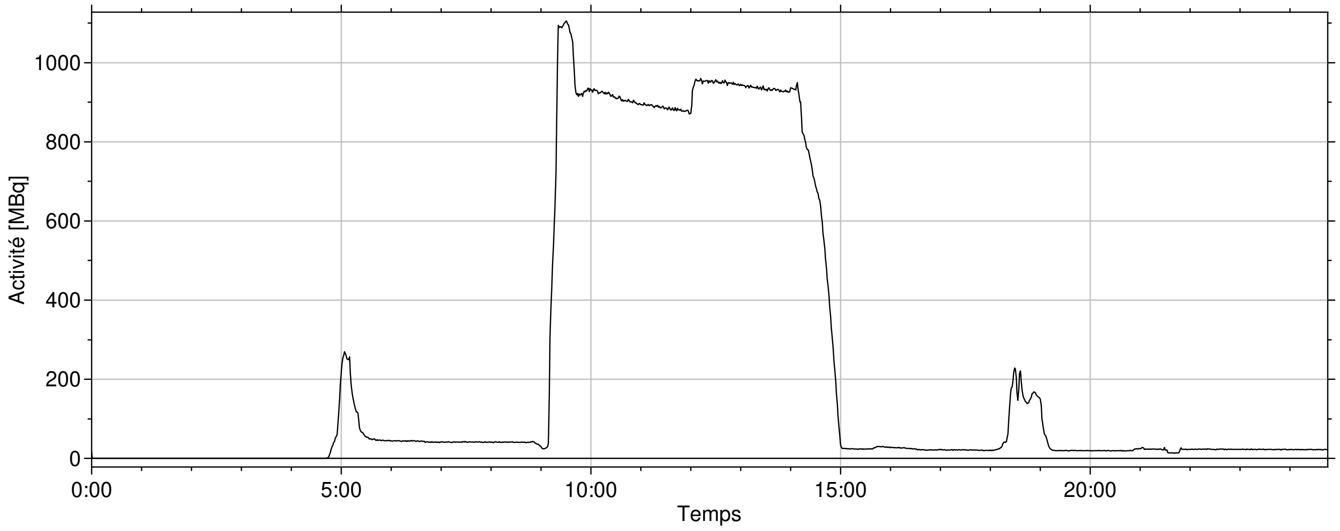

Gaia–Détecteur de la rampe 1

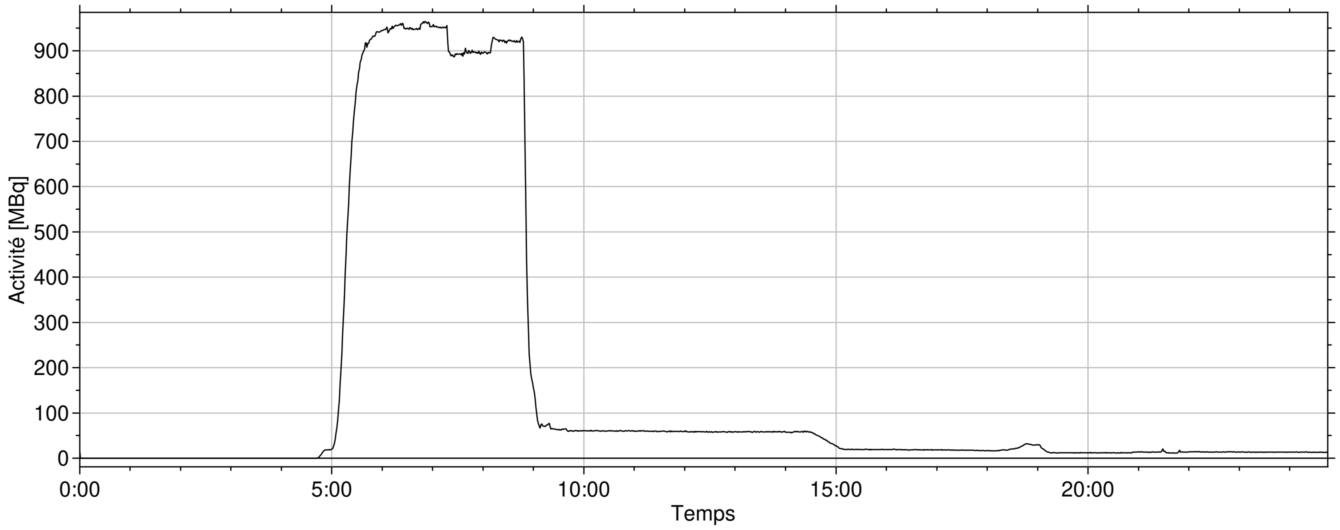

Gaia–Détecteur de la rampe 2

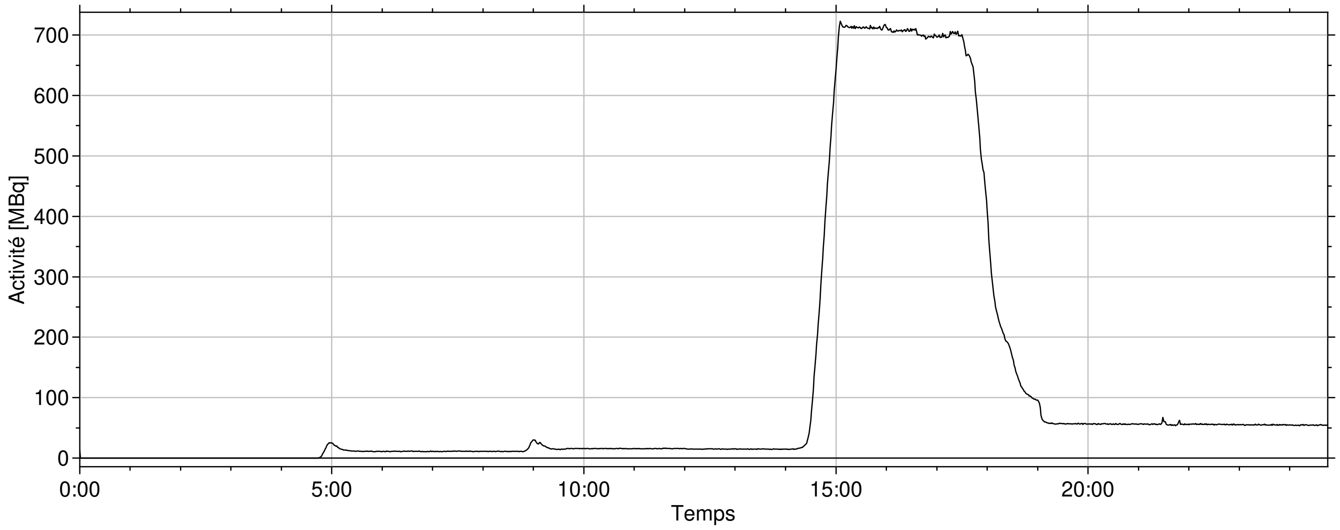

Luna–Position releveur

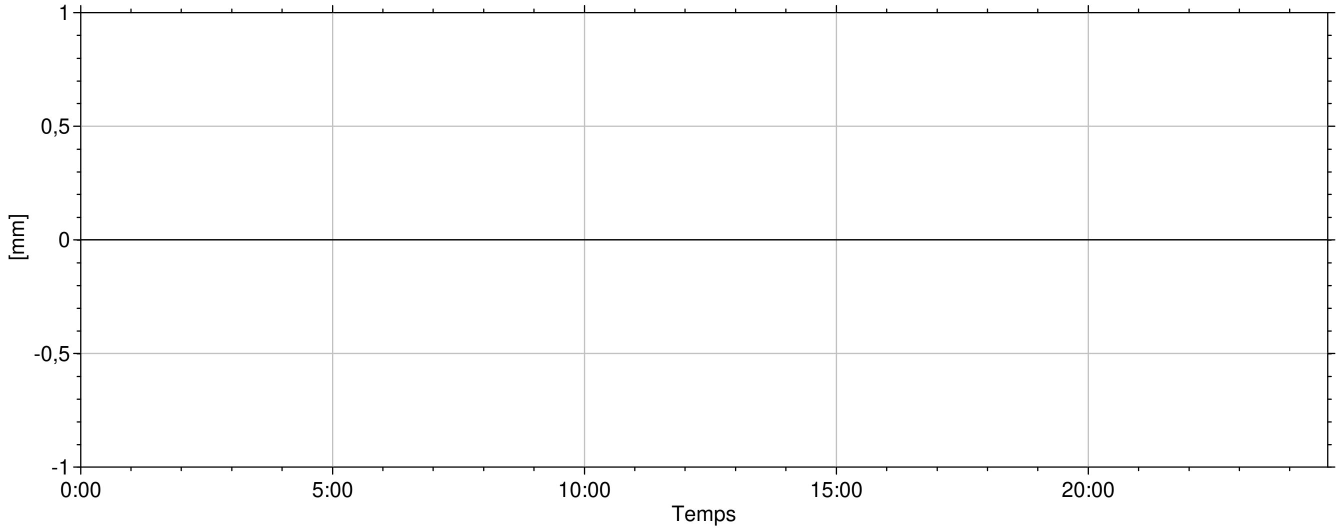

Luna–Température du four

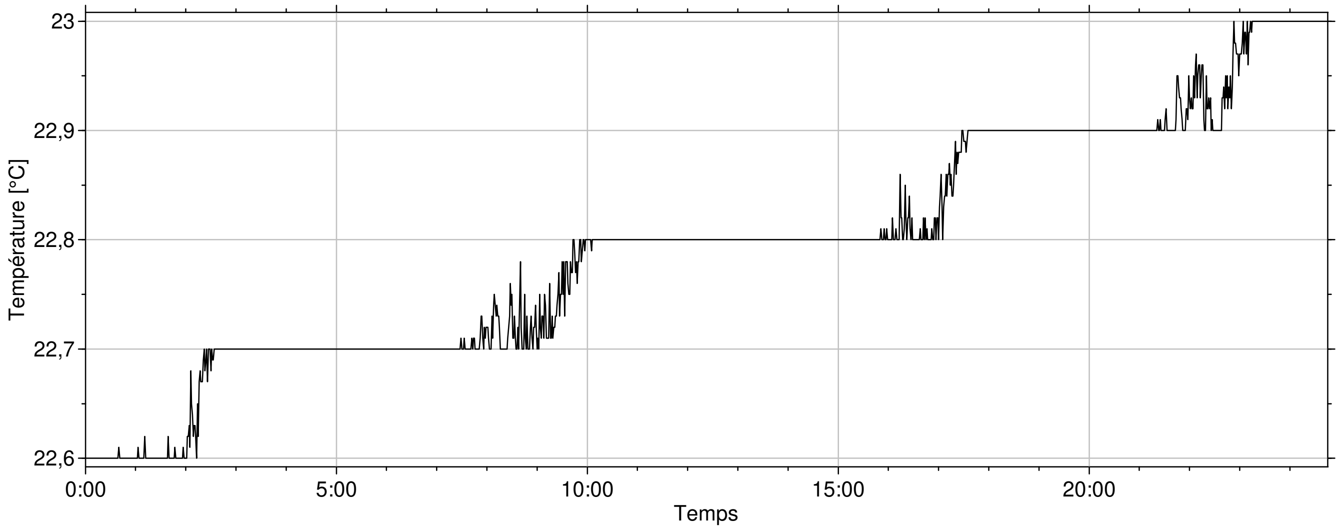

Luna–Puissance du four

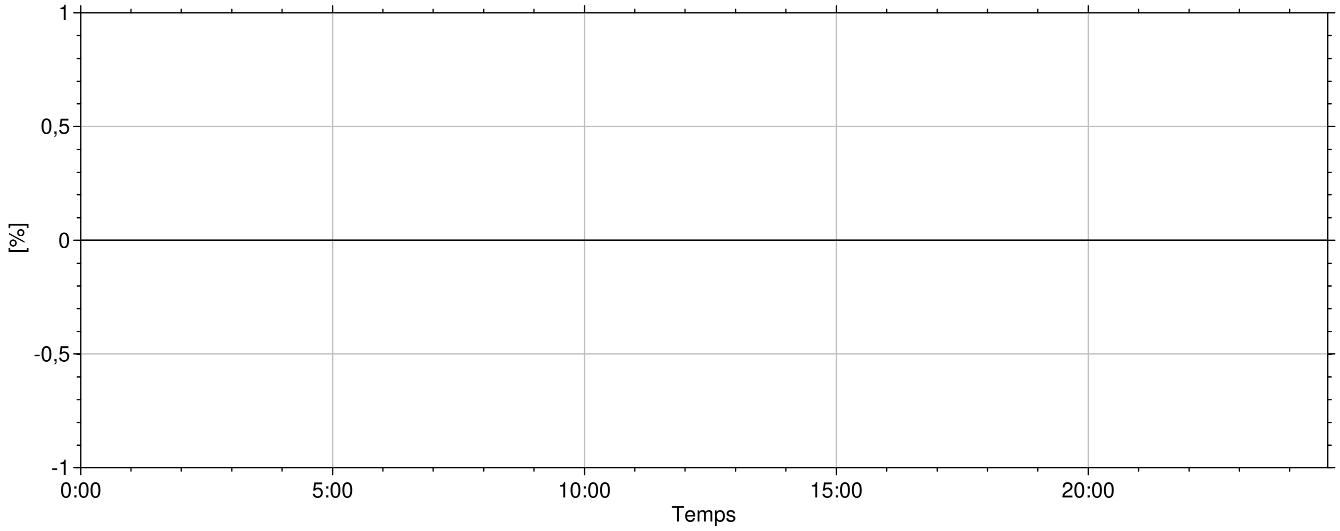

Luna–Détecteur du réacteur

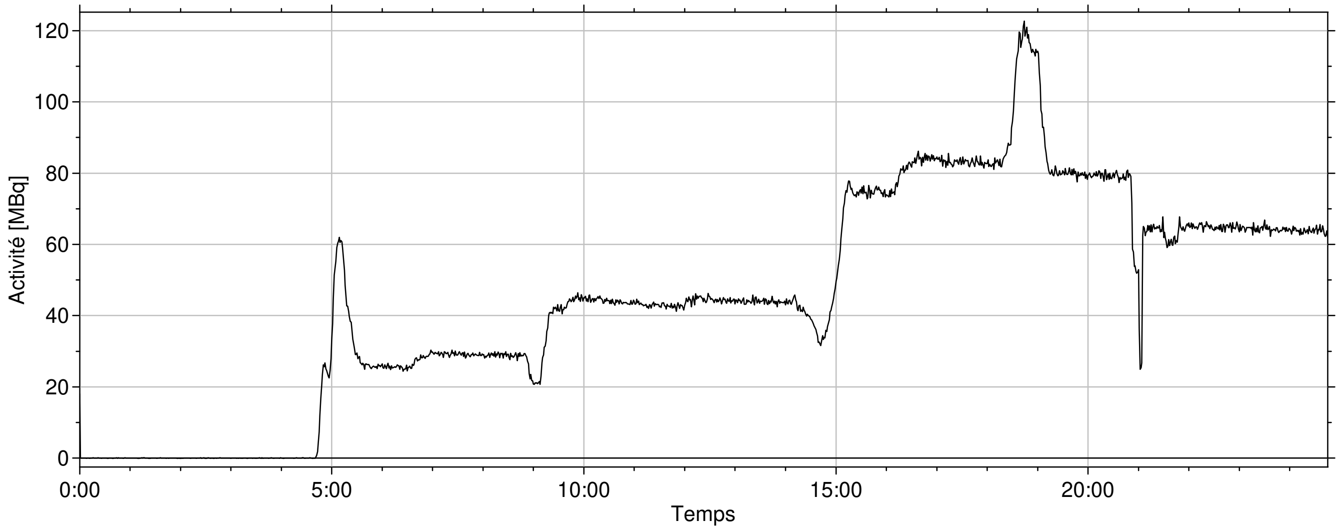

Luna–Tension du détecteur

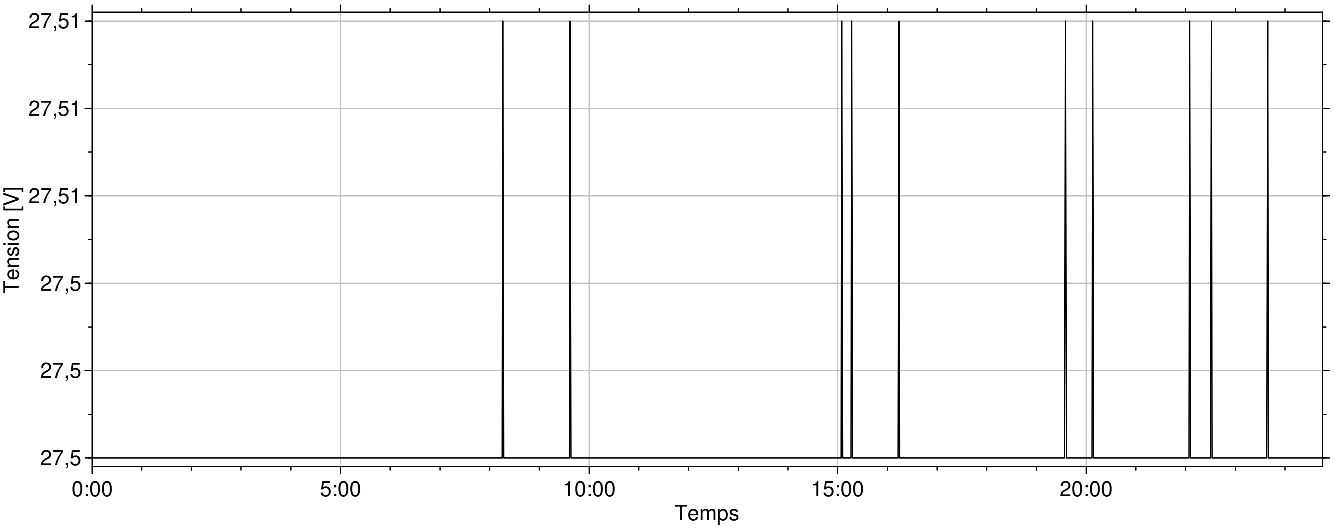

Supplement: Supplementary file 3 — Additional file 3.. GMP-conditions synthesis report N°3. This document shows all details dealing with technique parameters of the Gaia/Luna Elysia-Raytest module during the third GMP-condition synthesis. [file 41181_2023_187_MOESM3_ESM.pdf]
